# Brain training improves recovery after stroke but waiting list improves equally: A multicenter randomized controlled trial of a computer-based cognitive flexibility training

**DOI:** 10.1371/journal.pone.0172993

**Published:** 2017-03-03

**Authors:** Renate M. van de Ven, Jessika I. V. Buitenweg, Ben Schmand, Dick J. Veltman, Justine A. Aaronson, Tanja C. W. Nijboer, Suzanne J. C. Kruiper-Doesborgh, Coen A. M. van Bennekom, Sascha M. C. Rasquin, K. Richard Ridderinkhof, Jaap M. J. Murre

**Affiliations:** 1 Department of Psychology, University of Amsterdam, Amsterdam, The Netherlands; 2 Department of Medical Psychology, Academic Medical Center, University of Amsterdam, Amsterdam, The Netherlands; 3 Department of Psychiatry, VU University Medical Center, Amsterdam, The Netherlands; 4 Department of Research and Development, Heliomare Rehabilitation Center, Wijk aan Zee, The Netherlands; 5 Center of Excellence in Rehabilitation Medicine, Brain Center Rudolf Magnus, University Medical Center Utrecht, and De Hoogstraat Rehabilitation, Utrecht, The Netherlands; 6 Department of Experimental Psychology, Utrecht University, Utrecht, The Netherlands; 7 Amsterdam Rehabilitation Research Center, Reade, Amsterdam, The Netherlands; 8 Adelante-Zorggroep, Maastricht University, Caphri, Rehabilitation Medicine, Maastricht, The Netherlands; 9 Amsterdam Brain & Cognition (ABC), University of Amsterdam, Amsterdam, The Netherlands; University of Glasgow, UNITED KINGDOM

## Abstract

**Background:**

Brain training is currently widely used in an attempt to improve cognitive functioning. Computer-based training can be performed at home and could therefore be an effective add-on to available rehabilitation programs aimed at improving cognitive functioning. Several studies have reported cognitive improvements after computer training, but most lacked proper active and passive control conditions.

**Objective:**

Our aim was to investigate whether computer-based cognitive flexibility training improves executive functioning after stroke. We also conducted within-group analyses similar to those used in previous studies, to assess inferences about transfer effects when comparisons to proper control groups are missing.

**Methods:**

We conducted a randomized controlled, double blind trial. Adults (30–80 years old) who had suffered a stroke within the last 5 years were assigned to either an intervention group (n = 38), active control group (i.e., mock training; n = 35), or waiting list control group (n = 24). The intervention and mock training consisted of 58 half-hour sessions within a 12-week period. Cognitive functioning was assessed using several paper-and-pencil and computerized neuropsychological tasks before the training, immediately after training, and 4 weeks after training completion.

**Results and conclusions:**

Both training groups improved on training tasks, and all groups improved on several transfer tasks (three executive functioning tasks, attention, reasoning, and psychomotor speed). Improvements remained 4 weeks after training completion. However, the amount of improvement in executive and general cognitive functioning in the intervention group was similar to that of both control groups (active control and waiting list). Therefore, this improvement was likely due to training-unspecific effects. Our results stress the importance to include both active and passive control conditions in the study design and analyses. Results from studies without proper control conditions should be interpreted with care.

## Introduction

Approximately 60% of stroke survivors show cognitive impairments which often persist in the chronic phase after stroke [1,2]. Executive impairments, in particular, have a large impact on everyday life and may predict poor cognitive recovery after stroke [3,4], making efforts to improve these functions highly relevant. Computer-based training approaches may complement existing rehabilitation programs. They have the advantage that they can be exercised at home, thus facilitating intense and repeated practice, a key element for restitution-based rehabilitation. The aim of restitution training is not to master compensational strategies, but restoration of impaired functions through stimulation. It is very difficult, if not impossible, to distinguish whether improvements in cognitive functioning are due to restitution of the function or to the use of implicitly learned strategies. Still, it is important to evaluate whether mere retraining of cognitive functions can result in improved cognitive functioning, because restitution-based therapy has been effective in the domains of motor function, language, and vision (e.g., [5,6]).

So far, the evidence for the effectiveness of computer-based training in improving executive functioning after acquired brain injury is inconclusive. Several studies have reported improvements in tasks similar to the training (near transfer effects) as well as improvements in tasks that differ from the training (far transfer effects). Most of these studies, however, suffered from methodological limitations [7]. First, most studies lacked a control condition or included only passive (non-treated) control conditions. In studies that included active control (i.e. mock training) groups, computer-based training failed to outperform mock training [8,9]. Without an active control condition, positive training effects may well result from nonspecific elements such as spontaneous recovery, test-retest effects, or the Hawthorne effect (i.e., the effect of merely participating in a scientific study, entailing expectancy, personal attention, motivation, et cetera; [10,11]). Without a waiting list control condition, improvement in both training groups could be due either to both programs being effective or to nonspecific elements. Thus, both an active control condition and a waiting list control condition should be included to control for all training-unspecific effects. Second, most studies did not adjust statistically for multiple testing despite including a large number of outcome measures, and are, therefore, prone to type 1 error (i.e., report of positive results where there are none). However, there are some studies with healthy individuals that did correct for multiple testing (e.g., [12]). Third, the relation between study outcomes and training task progression was often not investigated, so it remains unclear to what extent functional improvements were related to the training. Furthermore, training duration was generally short (median = 15.6 hours), and sample sizes were relatively small (median = 16; [7]).

A further methodological issue is that previous training studies may not have targeted relevant and/or process-pure cognitive functions or their underlying neural mechanisms. Three major components of executive functions have been discerned: (1) control of one’s behavior, including inhibition of strong but inappropriate responses, (2) mental set shifting (i.e., changing from one set of task rules to another), and (3) information updating [13]. Training studies that did reveal reliable far transfer in healthy elderly commonly involved rapid task switching as a key ingredient of training [14], thereby targeting mental set shifting, which is at the core of executive functioning.

The aim of the current study was to test the hypothesis that three months of computer-based, commercially available, cognitive flexibility training improves executive functioning after stroke, while accounting for the above-reviewed methodological issues. We additionally conducted within-group analyses similar to those used in previous studies, to assess inferences about transfer effects when comparisons to proper control groups are missing. Participants trained five times per week half an hour for 12 weeks, which we expected to suffice to trigger restitution-based recovery of executive functions. The intervention training included rapid task switching. Difficulty of tasks was adapted individually to the performance of participants [14]. An active control group (i.e. mock training) and a waiting list group were included to control for nonspecific effects. We expected that the cognitive flexibility training would result in more pronounced transfer effects on executive functioning compared to the mock training, and that the performance of the waiting list group would not change over time.

## Materials and methods

A detailed description of the design, training tasks, and outcome measures of this study has been published previously [15].

### Participants

Participants were recruited from six Dutch rehabilitation centers and patient societies (April 2013—March 2015; last follow-up measurement in November 2015). They were included when they had had a stroke 3 months to 5 years ago, were between 30 and 80 years old, and (had) received rehabilitation therapy as inpatient or outpatient. Participants were required to have cognitive *impairments* after stroke (as testified by medical records), with cognitive *complaints* still present at study entry. Finally, participants were required to be able to work with the computer and have daily access to a computer with Internet connection.

Exclusion criteria were presence of neurodegenerative disease; epilepsy; serious psychiatric illness; any disease other than stroke that results in severe cognitive impairments; drug or alcohol dependency; severe color blindness, aphasia, neglect, or computer fear; disabling vision or auditory problems; and diagnosed learning disability. Furthermore, participants who were not mentally or physically fit enough to be able to complete 12 weeks of training were excluded. Finally, those who were not able to understand the training instructions or who could not execute the training due to any other unforeseen reason, after instructions or after the first training week, were excluded.

**Fig 1:**
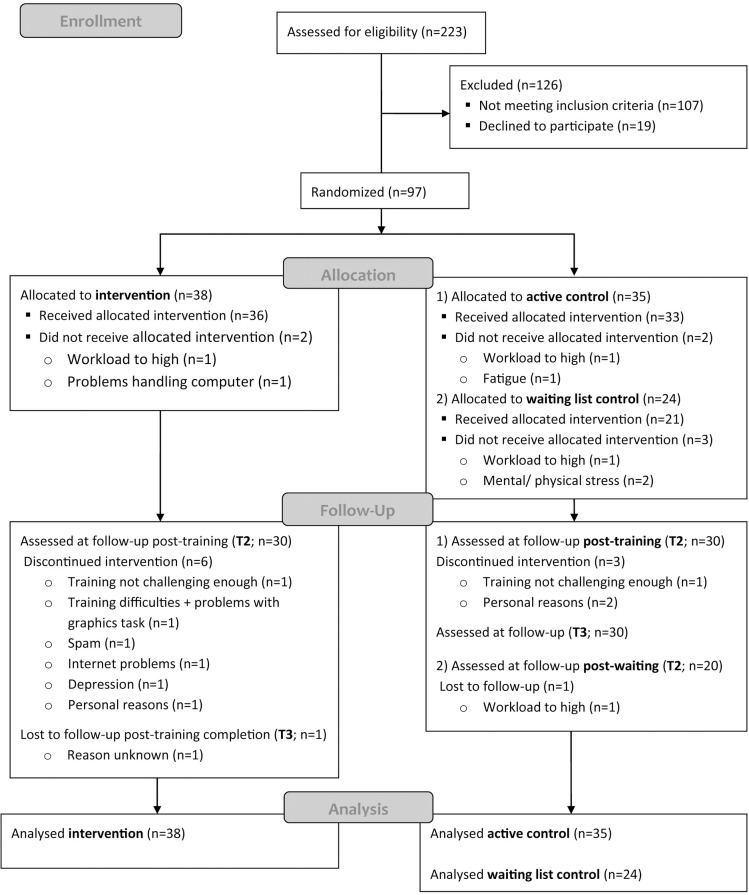
Consolidated Standards of Reporting Trials (CONSORT) flow diagram. T2 = post-training; T3 = 4 weeks after training completion.

A priori sample size calculations were based on our aim to detect at least large group effects [16] between two groups with one outcome measure. With a power of .80 and an alpha of .05 (one-tailed), this effect would be revealed in univariate analyses with a minimal sample size of 20 per group. As a switch training in healthy elderly resulted in an effect size *d* = 0.40 [17] we aimed to maximally include 138 participants, resulting in 3 x 40 while taking into account a 15% attrition rate. Inclusion would stop at this number or when the recruitment period was over. A schematic overview of the participant flow can be found in Fig 1.

### Experimental design

The study was a prospective multicenter, double-blind, randomized controlled study (RCT). Participants were assigned randomly to the intervention group, the active control group, or to the waiting list group by randomization software (Minimpy; [18]). This assured minimal differences between groups in time since stroke (post-acute versus chronic), level of computer experience (<0.5, 0.5–4, >4 hours), age (30–49, 50–59, 60–69, 70–80), education (primary or lower secondary, middle secondary, higher, university), cognitive screening scores (Telephone Interview for Cognitive Status, TICS [19]: <33, 33–37, >37), and sex (male, female). Participants were not informed that one of the training programs was a mock training. Instead they were told that the study aimed to compare two types of computer-based cognitive training programs. The groups were coded by the research coordinator such that the assessors were blind to which training condition the participant was assigned. The waiting list group was added during the course of the study (see S1 File). At that moment, 25 participants were included in the intervention group and 28 in the active control group.

Computer tasks were administered online at baseline (T0), after 6 weeks of training or waiting (T1), after training completion or after 12 weeks of waiting (T2), and (for the training groups) 4 weeks after training completion (T3). Conventional neuropsychological tasks tapping several cognitive domains were administered at T0 and T2. In addition, brain MRI scans were obtained at T0 and T2 in a subset of the sample and several questionnaires were administered at all time-points, results of which will be presented elsewhere.

The study was approved by the ethical review board of the University of Amsterdam (i.e., Commissie Ethiek voor de Afdeling Psychologie; approved December 2012) and by the medical ethical review board of the VU University Medical Center, Amsterdam (i.e., the Medisch Ethische Toetsingscommissie Vrije Universiteit Medisch Centrum; approved July 2013, amendment approved May 2014). The study is registered before study commencement as Training Project Amsterdam Seniors and Stroke (TAPASS) with the Central Committee on Research Involving Human Subjects Register NL4468502913 (www.toetsingonline.nl). Additionally, to fulfill the World Health Organization Registry criteria it was also registered with the Netherlands National Trial Register NTR5174. The authors confirm that all ongoing and related trials for this intervention are registered.

### Procedures

Whenever participants indicated that they wanted to participate in the study, they were asked to sign an online informed consent form and complete an online screening questionnaire and a cognitive screening by phone (TICS) to assess inclusion and exclusion criteria. After randomization (T0), participants gave written informed consent and performed neuropsychological and computer tasks administered by two junior psychologists trained and supervised by a neuropsychologist at the University of Amsterdam. The assessor of neuropsychological tasks was blind to the training allocation of the participant. After task administration, he/ she registered which training condition they thought (i.e., guessed) the participant was in, to check whether they were truly blind. Note that the person administrating the computer tasks and training instructions was not blind to training allocation. In addition, participants performed several computer tasks online at home (see [15] for a detailed description).

Participants in the training groups trained at home five times per week during half an hour for 12 weeks. The training program consisted of 58 training sessions (29 hours of training). A daily log was completed before and after each training session. A trained student contacted the participant by phone to ask about their training progression and an automatic training reminder was sent by e-mail whenever participants did not train for two days that week. The waiting list group received care as usual which most often meant they did not receive any treatment; they were not contacted by phone. After 12 weeks of either training or waiting (T2), participants came back to the university to perform the same tasks as before (T0). After completing these tasks, the waiting list group started the intervention training.

The online tasks were also administered after 6 weeks of waiting or training (T1). Four weeks after training completion (T3), the online tasks were repeated among training groups to measure long-term effects of the training.

### Intervention materials

Both computer training programs were carried out at home via a preexisting brain training website, www.braingymmer.com. The training tasks (games) were professionally programmed to be stimulating. Based on a pilot study, we adjusted elements such as the time allowed to complete a task to stroke survivors and to the elderly population. Individual feedback was given after each task based on a three-star rating scale and at the end of each session. During the workout, the next task was automatically selected and presented to the participant. Participants were thus not free to navigate the website and select their own tasks. They were, however, able to select the level of difficulty where a higher level became available as soon as one out of three stars was achieved. The training duration of 12 weeks with five half-hour sessions per week was based on previous training studies from which we concluded that a minimum training period of three months was required. Moreover, we asked stroke survivors and healthy elderly what they thought was an achievable time investment in addition to their everyday life activities.

#### Cognitive flexibility training

The intervention training consisted of nine tasks in the cognitive domains of working memory, attention, and reasoning. To stimulate cognitive flexibility, tasks from the same cognitive domain were never performed successively. In the first week, each task was performed for 10 minutes in order to get to know the task (i.e., three tasks per session). After that, sessions included 10 tasks of 3 minutes each to challenge cognitive flexibility. The degree of difficulty of the task was adapted to the participant’s performance; they were asked to continue to the next level as soon as they received two or three out of three stars. All tasks consisted of 20 levels.

#### Mock training

The mock training consisted of four tasks that were not expected to train executive functions because they did not involve updating, set shifting, or inhibition. Each session consisted of three tasks. Thus, participants only switched to the next task after 10 minutes. The tasks were not adaptive; participants were asked to train at the same level for one or two weeks before they were allowed to go to the next level. The instructors asked participants not to train beyond level nine. However, some participants disregarded this and trained at higher levels anyway.

### Outcome measures

#### Primary outcomes

For the primary analyses, executive functioning was measured with several neuropsychological tasks and one computer task (see Table 1 for outcome measures used per task). These included the number-letter switching condition of the Trail Making Test (TMT) from the Delis-Kaplan Executive Function System (D-Kefs; [20]), category fluency [21] and letter fluency tasks [22], an online version of the Tower of London (ToL, based on [23]), and Letter-Number Sequencing (LNS, Wechsler Adult Intelligence Scale III-NL; [24]). Raw scores were corrected for demographics at baseline assessment based on norm scores where available. None of the outcome measures was used in the training program, thus all measured transfer effects.

**Table 1:** Tasks used for the cognitive domains.

Cognitive domain	Task	Outcome measure	
*Primary outcome measures*			
Executive functioning	- D-Kefs TMT number-letter switching condition [20]	Age-corrected z-scores of time to completion	
	- Category fluency [21]	Number of words mentioned within one minute (average of two categories)	
	- Letter fluency [22]	Education-corrected z-score of number of words mentioned within one minute (three different starting letters)	
	- Tower of London* [23]	Number of moves—minimal required moves to solve the ToL‡. N.B. Maximal possible moves score per item was 20 and unsolved items were scored with 20	
	- Letter-Number Sequencing [24]	Age-corrected z-scores of total number of correct items	
*Secondary outcome measures*			
Cognitive flexibility	- Switch-task [25]	1) Switch cost RT: RT on switch—no switch trials in ms‡ 2) Switch cost accuracy: accuracy on no switch—switch trials‡	
	- Dual-task	1) Dual cost RT: RT on speeded response of the dual trials—no switch trials in ms‡ 2) Dual cost accuracy: accuracy on no switch—speeded trials‡	
	- Category fluency switch condition	Average number of words mentioned in two no-switch categories minus words mentioned in switch condition: switch cost = (category 1 + category 2)/2—switch category)‡	
	- TMT B*† (NeuroTask BV)	Time taken to complete TMT B in sec (connecting letters and numbers in alternating order) ‡	
Attention	- TMT A*† (NeuroTask BV)	Time taken to complete TMT A in sec (connecting numbers) ‡	
	- Paced Auditory Serial Addition Task (PASAT; [26])	Percentage correct on condition 2.8 and 3.2	
	- Digit-Symbol-Coding (DSC; [24] and NeuroTask BV*)	For T0 and T2 analyses: items correct within two minutes of the paper version	
			For T0 and T3 analyses: items correct within two minutes of the online version
Memory	**-** Rey’s Auditory Verbal Learning Test (RAVLT; [27])	1) Direct: sex-, age- & education-corrected z-scores of total amount of words remembered on 5 trials 2) Delayed: sex-, age- & education-corrected z-scores of total amount of words remembered during delayed recall corrected for direct total score	
Working Memory	- N-back† [28]	Percentage correct on the 2 back—percentage correct on 0 back	
	- Blokkenreeksen (NeuroTask BV); online modified version of Corsi task*†	The longest correctly reproduced array	
Reasoning	- Raven Progressive Matrices* [29]	Total number of correct responses on 20 items	
	- Shipley Institute of Living Scale-2* [30]	Total number of correct responses	
Psychomotor speed	- D-Kefs TMT motor speed condition [20]	Age-corrected z-scores of time to completion	
	- Mouse skills tasks*† (NeuroTask BV)	1) Drag and drop skill: average RT for all moves in sec‡ 2) Drag skill grid: total time spent on task from first click until last drop in sec‡ 3) Click skill: total time from first click to till last click in sec‡	
Inhibition	- Stop-signal task †	SSRT (i.e., average time needed to inhibit a go response on stop trials) in ms‡	

*Note*. D-Kefs = Delis-Kaplan Executive Function System; TMT = Trail Making Test

* = Online measure

† = See van de Ven (2015) for a task description,

‡ = Recoded such that higher scores represent better performance (e.g., multiplied by -1).

#### Secondary outcomes

Objective cognitive functioning was assessed for the following domains: cognitive flexibility, attention, verbal memory, working memory, reasoning, psychomotor speed, and inhibition. Most domains consisted of multiple tasks (see Table 1 for tasks and outcome measures used per domain). The operation span task was not used for analyses, because online task presentation became unreliable due to changes in the Flash plugin in the most used internet browser. All scores were recoded in such a way that higher scores represent better performance. For details about data preparation of the primary and secondary outcomes, see S1 File.

#### Training performance

For both experimental groups, training performance was assessed based on task level and highest score obtained per level. Per task, the obtained high-scores for each level were converted into a percentage of the maximal possible score and added up to a total task score. The intervention training consisted of three domains and the domain score was based on the average task score of the tasks within that domain. Finally, a total score was calculated by taking the average of the three domain scores (i.e., the average score of all tasks). The mock training consisted of four tasks and the total score was based on the average of these tasks. Scores could range from 0 to 2000 (i.e., maximum 100 per level).

### Statistical analysis

Primary analyses of transfer effects were performed with one repeated-measures MANOVA. The dependent variables were time to completion on the switch condition of the D-Kefs TMT, number of words mentioned during the category fluency and the letter fluency tasks, square root transformation of the number of steps of the ToL, and score on LNS. The independent variable was group (intervention, mock training, and waiting list control group). Time-points in this model were before (T0) and after training or waiting period (T2). Post-hoc univariate ANOVAs were performed when the time effect of the MANOVA was significant. P-values that do not survive Bonferroni-Holm correction are reported.

Secondary analyses were performed in a similar way, i.e. by a single repeated-measures MANOVA, with inhibition and composite scores of cognitive flexibility, attention, verbal memory, working memory, reasoning, and psychomotor speed as dependent variables. A composite score was calculated per domain by calculating the average z-score based on the mean and standard deviation of all participants at T0. However, if norm scores were available, demographically-corrected z-scores were used instead. Both primary and secondary analyses were repeated with age, education, and time since stroke as covariates to explore the influence of these variables on training effects. We performed these analyses because they were originally planned. Due to absent correlations between the covariates and the outcome measures and a lack of statistical power, the outcomes are not statistically reliable (results can be found in S1 File).

Whenever the training resulted in a significant improvement of the dependent variables that were additionally measured at T1 (after 6 weeks of training) and T3 (follow-up 4 weeks after training completion), the time-points T1 and T3 were added to the model. This was done to determine whether the training was already effective after 6 weeks of training, and to assess whether training effects would persist after the training.

Blinding for training assignment of assessors and participants was checked with a binomial test. Training performance differences between groups were analyzed with Mann-Whitney tests because the training scores were not normally distributed. The dependent variable was the difference score, which was calculated by subtracting the average training score after the first time each task was performed (10 minutes per task; at T0) from the final average training score (at T2). The independent variable was group (intervention and mock training). The relation between training improvement and performance change on outcome measures was examined with Pearson’s correlation (statistically tested one-tailed).

Exploratory Student paired t-tests were performed in both the training groups and the waiting list group with all outcome measures at T0 and T2 as dependent variables in order to compare our results with previous training studies. In addition, Bayesian independent samples t-test was performed with JASP (Version 0.7.5.5; Computer software) to explore whether the evidence was in favor of H0 (data are from the same group) or H1 (data come from two groups; training groups ≠ waiting list group). Finally, to examine whether the training had an effect on overall cognition, a repeated-measures ANOVA was conducted with a composite score of all outcome measures as the dependent variable and group (intervention, mock training, and waiting list control group) as independent variable. Norm-corrected z-scores were used where available. For the remaining tasks, z-scores were calculated based on the mean and standard deviation at T0.

All analyses were performed as intention-to-treat analysis, including all participants who started the study. Additionally, analyses were rerun as per-protocol analysis. In these latter analyses, only participants who completed the training according to protocol (e.g., completed at least 50 sessions) and who performed the tasks at T2, were included in the analyses.

Outliers in the (transformed) raw data were detected by Grubbs’ Extreme Studentized Deviation test [31] and were replaced with the closest value of the other participants. Missing values that were due to the participant (e.g., too tired to complete the task, or incidentally incapable of performing the task altogether) were substituted with the lowest observation of the group at that time-point for that task, or for the D-Kefs TMT with *z* = -3 as this is the lowest score possible for that task. Data that were missing but not due to the participant (e.g., caused by technical problems) were substituted based on the last observation carried forward. In cases where the baseline score was missing, last observation carried backwards was used, such that the data from the closest time-point after baseline was used. If both time-points were missing, the average of the group was used. Note that in this way substituted data were conservative. The results, therefore, more likely reflect an underestimation than an overestimation. Multivariate outliers were only replaced if an explanation could be found for the extreme values. Analyses were run with and without outliers. All reported results are without univariate outliers and with multivariate outliers, but if results differed, both analyses were reported.

Normality was checked with Shapiro-Wilk test and by evaluating skewness and kurtosis. SPSS version 19 (IBM; Armonck, USA) or a later version was used. P-values of .05 or lower (two-tailed if not mentioned otherwise) were considered significant.

## Results

### Pre-training

Out of 223 potential participants who were screened, 97 passed all in- and exclusion criteria and were included in the final analyses (see Fig 1 for participant flowchart including drop-out reasons). Prior to training, the three groups did not differ in age, educational level, sex, time since stroke, or baseline cognitive functioning except for attention (see Table 2 for scores and statistics). The intervention group had significantly higher baseline scores on the attention composite (*p* < .01) and reported significantly higher levels of fatigue (*p* < .01) than the active control group, but not waiting list group.

**Table 2:** Mean (standard deviation) of demographic variables and baseline (T0) outcome measures.

	Intervention group	Active control group	Waiting list group		
	(n = 38)	(n = 35)	(n = 24)	Sign.	
Age (M/median (SD))	57.0/55.0 (9.1)	60.9/ 62.0 (7.5)	61.2/ 60.5 (9.0)	.08	
Education (M/median (SD, range))	5.6/6 (1.1, 2–7)	5.6/6 (1.1, 2–7)	5.5/6 (1.3, 2–7)	.95	
Sex (% male)	63	66	79	.39	^d^
Time since stroke (in months; M/median (SD, range))	28.3/28.0 (16.4, 4.6–59.3)	28.3/29.0 (14.4, 4.1–51.5)	29.1/27.3 (17.0, 5.4–61.1)	.98	
TICS (M/median (SD, range))	34.6/35 (2.1)	34.1/34 (2.8)	34.2/35 (2.4)	.63	
Cogn. Rehab. during study (% yes)	5	14	12	.42	^d^
Non cogn. rehab. During study (% yes)	34	40	24	.50	^d^
*Baseline scores of primary outcome measures*					
- D-Kefs TMT (number-letter switching)	0.1 (1.1)	-0.6 (1.3)	-0.3 (1.2)	.08	
- Letter Number Sequencing	0.1 (1.1)	0.0 (1.2)	-0.3 (1.2)	.52	
- Letter fluency	-0.4 (1.2)	-0.7 (1.2)	-0.8 (1.0)	.22	
- Category fluency	20.1 (6.0)	18.8 (7.4)	17.6 (3.7)	.29	
- Tower of London	-5.5 (1.8)	-5.3 (2.4)	-5.5 (2.0)	.94	
*Baseline scores of secondary outcome measures*					
- Cognitive flexibility composite	0.0 (0.4)	0.0 (0.6)	0.0 (0.4)	.72	
- Attention composite	0.1 (0.8)	-0.5 (0.8)	-0.3 (0.7)	**.01**	
- Verbal memory composite	47.9 (9.7)	48.3 (9.3)	48.4 (9.9)	.98	
- Working memory composite	0.1 (0.8)	-0.1 (0.8)	0.1 (0.8)	.54	
- Reasoning composite	0.0 (0.8)	0.0 (0.9)	-0.1 (1.0)	.90	
- Psychomotor speed composite	0.1 (0.6)	0.0 (0.8)	0.0 (0.7)	.56	
- Inhibition	-298 (43)	-291 (68)	-289 (81)	.83	
CIS-F	39.4 (11.7)	30.9 (12.5)^a^	34.3 (12.5)	**.01**	
HADS D	6.1 (3.8)	5.3 (3.6)^b^	5.3 (2.7)^c^	.58	^ ^

*Note*. All scores are z-scores except for semantic fluency (words mentioned), Tower of London (reversed score of extra moves required), and inhibition (ms). P-values are based on ANOVA (if not mentioned otherwise). Bold values are considered significant. Education was based on a 7-point scale (from 1 = unfinished primary school to 7 = university). Sign. = significance; TICS = Telephone Interview for Cognitive Status; Cogn. Rehab. = cognitive rehabilitation; D-Kefs TMT = Delis-Kaplan Executive Function System Trail Making Test; CIS-F = Checklist Individual Strength- Fatigue subscale; HADS D = Hospital Anxiety Depression Scale—Depression

^a^ = n = 34

^b^ = n = 33

^c^ = n = 20

^d^ = p-value based on χ^2^.

The blinding for training assignment was confirmed. Assessors of the neuropsychological tasks did not guess the training condition significantly better than chance at baseline (T0: 30%; *p* = .30) and after training or waiting (T2: 42%; *p* = .11). The active control group was not informed about the existence of a mock training. More than half (66%) of the active control group thought they had received an intervention training compared to 89% of the intervention group. Moreover, the training groups did not significantly differ with respect to motivation during training, perceived difficulty of or interest in the training, number of workouts completed, or drop-out rate. This suggests that the active control group did perceive their training similarly to the intervention group.

### Training tasks

Thirty-six participants started the intervention training and 33 started the mock training. The average number of training sessions completed was 48.4, which equals 24.2 hours. This did not differ significantly between the intervention training group and the active control group (*t*(71) = .44, *p* = .66). From the participants who completed the post-training assessment (T2; *n*_intervention_ = 29, *n*_active control_ = 30), the average number of sessions completed was 56.8 (i.e., 28.4 hours). Both training groups improved on the training tasks (see S1 Fig) and improvement per task did not differ significantly between groups (Mann–Whitney *U* = 628.5, *n*_intervention_ = 36, *n*_active control_ = 33, *p* = .68).

The intervention training was intended to be more adaptive than the mock training. However, the degree of adaptiveness was compromised in 83% of the active control group and 17% of the intervention group participants. First, even though the active control group had been instructed to stay below level 10, 83% of the active control participants continued at levels higher than nine. They did so in on average 10% of their training time compared to 14% in the intervention group, which was allowed to train until level 20. Thus, the mock training was more adaptive than originally planned, which may mitigate the difference between intervention and mock training. However, because the intervention training included rapid task switches and tasks were focused on executive functions it was still believed to be superior to the mock training. Second, in the intervention group, five participants (17%) were slightly less challenged in the last weeks of the training because they reached the highest level and score possible on *one of the nine* tasks. Though not likely, this may have yielded a small ceiling in training effects.

### Transfer effect of training

#### Executive functioning measures (primary outcome measures)

In the repeated-measures MANOVA with the five main executive functioning outcome measures (see statistical analysis section), all three groups improved significantly over time (*F*(5,90) = 7.85, *p* < .001, with partial eta squared effect size (*ɳ*_*p*_^*2*^*)* = .30), but there was no group*time interaction (*F*(10,182) = 0.78, *p* = .65, *ɳ*_*p*_^*2*^ = .04; see Table 3). Time effects were shown for the D-Kefs TMT (*p* < .001, *ɳ*_*p*_^*2*^ = .20), LNS (*p* < .01, *ɳ*_*p*_^*2*^ = .07), and ToL (*p* < .01, *ɳ*_*p*_^*2*^ = .08), but the intervention training did not result in larger improvements compared to either of the control groups. Several active control participants trained at higher levels than was allowed and five intervention group participants reached highest levels for two training tasks. Results did not change when these participants were left out.

**Table 3:** Mean (standard deviation) and repeated-measures MANOVA of the outcome measures.

								Group								Comparison					
	Intervention group (n = 38)					Active control group (n = 35)					Waiting list group (n = 24)					Time			Time*group		
measure	Pre-training		Post-training		Δ	Pre-training		Post-training		Δ	Pre-waiting		Post-waiting		Δ	F^α^	p-value	*ɳ*_*p*_^*2*^	F^α^	p-value	*ɳ*_*p*_^*2*^
*Primary*																F_(5,90)_	**<.001**	.30	F_(10,182)_	.65	.04
DKEF TMT (switch)	0.1	(1.1)	0.2	(1.0)	0.2	-0.6	(1.3)	-0.1	(1.2)	0.5	-0.3	(1.2)	-0.1	(1.2)	0.2		**<.001**	.20			
Letter Number Seq.	0.1	(1.1)	0.2	(1.1)	0.1	0.0	(1.2)	0.2	(1.1)	0.2	-0.3	(1.2)	0.0	(1.1)	0.3		**<.01**	.07			
Phonetic fluency	-0.4	(1.2)	-0.2	(1.3)	0.1	-0.7	(1.2)	-0.7	(1.3)	0.1	-0.8	(1.0)	-0.8	(0.8)	0.0		.20	.02			
Semantic fluency	20.1	(6.0)	19.9	(6.4)	-0.1	18.8	(7.4)	18.8	(6.9)	0.0	17.6	(3.7)	18.3	(4.2)	0.7		.61	.00			
Tower of London	-33.4	(22.9)	-25.6	(15.6)	7.8	-34.0	(26.0)	-29.0	(21.5)	4.9	-34.2	(19.8)	-27.6	(14.4)	6.6		**<.01**	.08			
*Secondary*																F_(7,88)_	**<.001**	.41	F_(14,178)_	.69	.06
Cognitive Flexibility	0.0	(0.4)	0.1	(0.4)	0.1	0.0	(0.6)	-0.1	(0.5)	-0.1	0.0	(0.4)	0.0	(0.5)	0.0	0.1	.72	.00			
Attention	0.1	(0.8)	0.3	(0.9)	0.2	-0.5	(0.8)	-0.2	(0.9)	0.3	-0.3	(0.7)	-0.1	(0.8)	0.2	40.5	**<.001**	.30			
Verbal memory	-0.2	(1.0)	-0.2	(0.9)	0.1	-0.2	(0.9)	-0.3	(1.0)	-0.1	-0.2	(1.0)	-0.1	(1.1)	0.1	0.0	.83	.00			
Working memory	0.1	(0.8)	0.1	(0.7)	0.0	-0.1	(0.8)	-0.1	(0.6)	0.0	0.1	(0.8)	0.0	(0.8)	0.0	0.0	.89	.00			
Reasoning	0.0	(0.8)	0.2	(0.9)	0.2	0.0	(0.9)	0.2	(1.2)	0.1	-0.1	(1.0)	0.1	(0.9)	0.2	5.0	**.03**^**a**^	.05			
Psychomotor speed	0.1	(0.6)	0.4	(0.7)	0.3	0.0	(0.8)	0.3	(0.7)	0.3	0.0	(0.7)	0.2	(0.6)	0.1	41.0	**<.001**	.30			
Inhibition	-298	(43)	-285	(64)	13	-291	(68)	-299	(96)	-8	-289	(81)	-277	(56)	12	0.8	.37	.01			
Overall cognition	0.0	(0.4)	0.1	(0.4)	0.1	-0.1	(0.5)	0.0	(0.5)	0.1	-0.1	(0.4)	0.0	(0.4)	0.1	F_(1,94)_	**<.001**	.18	F_(2,94)_	.48	.02

Note. All scores are z-scores except for semantic fluency (words mentioned), Tower of London (reversed score of extra moves required), and inhibition (ms); Higher scores represent better performance; F was based on Pillai's Trace; Results were not affected by including outliers. Bold values are considered significant.

^a^ = becomes non-significant after Bonferroni-Holm adjustment

*ɳp*^2^ = partial eta squared (effect size); DKEF TMT = Delis-Kaplan Executive Function System Trail Making Test condition 4; Seq. = Sequencing.

#### Cognitive flexibility and other cognitive domains (secondary outcome measures)

In the repeated-measures MANOVA with the secondary composite measures, the performance of all three groups increased significantly over time (*F*(7,88) = 8.89, *p* < .001, *ɳ*_*p*_^*2*^ = .41), but there was no group*time interaction (*F*(14,178) = 0.77, *p* = .69, *ɳ*_*p*_^*2*^ = .06; see Table 3). Univariate repeated-measures ANOVAs revealed improvements over time (T2-T0) for attention (*p* < .001, *ɳ*_*p*_^*2*^ = .30), reasoning *(p* = .03, *ɳ*_*p*_^*2*^ = .05) and psychomotor speed (*p* < .001, *ɳ*_*p*_^*2*^ = .30), but the intervention training did not result in larger improvement compared to either of the control groups. The time effect for reasoning did not survive adjustment for multiple comparisons. Results were similar when analyses were repeated with univariate outliers, except for the time effect of reasoning which obtained trend-wise significance (*p* = .06, *ɳ*_*p*_^*2*^ = .04). As with the primary analyses, results did not change when analyses were run without participants for whom adaptiveness of the training was compromised.

#### Relation between improvement on training task and outcome measures

The improvement on training tasks did not correlate significantly with improvement on primary transfer tasks (*r* ranging from -.13 to .31). The only significant correlation was between the attention domain score of the intervention training and the improvement on the ToL (*r*(34) = .31, *p* = .03). For the secondary outcome measures the correlations were also low to moderate (*r* ranging from -.19 to .45). Significant correlations in the intervention group were found between improvements in attention training tasks and reasoning composite (*r*(34) = .31, *p* = .03); memory training tasks and attention composite (*r*(34) = .33, *p* = .02); and improvement of reasoning training tasks correlated with improvements in the attention composite, reasoning composite, and psychomotor speed composite (*r* = .35, *p* = .02; *r* = .32, *p* = .03; *r* = .28, *p* = .05; respectively). Mock training improvement only correlated significantly with attention composite improvement (*r*(31) = .45, *p* < .01).

#### Planned explorative analyses

**Fig 2:**
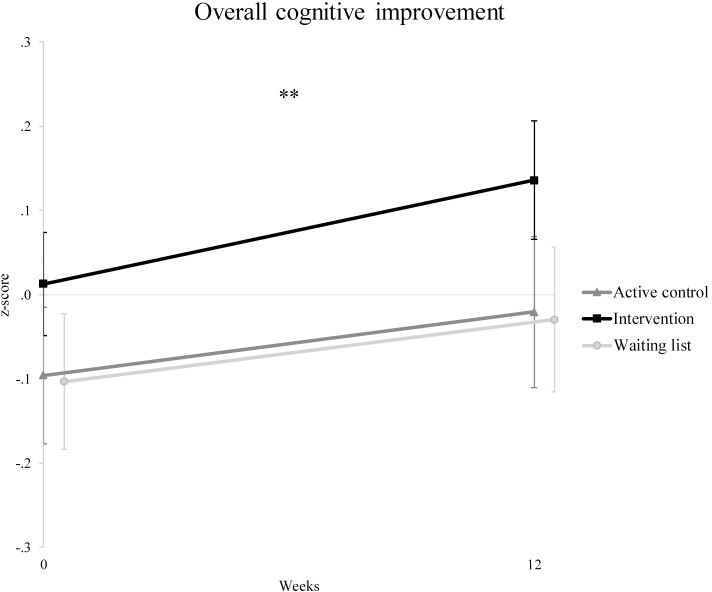
Overall cognitive improvement on all outcome measures combined into one composite measure. n_active control_ = 35, n_intervention_ = 38, n_waiting list_ = 24. ** = The time effect was significant (*p* < .001) without correcting for covariates. Groups did not differ significantly at baseline nor was there a significant group*time interaction. Error bars represent standard errors.

We performed a repeated-measures ANOVA to examine the training and waiting effect on overall cognition based on a composite score of all outcome measures, and obtained results similar to those above. There was a significant time effect (*F*(1, 94) = 20.8, *p* < .001, *ɳ*_*p*_^*2*^ = .18), indicating that the performance of all three groups improved. However, the group*time interaction effect was not significant (*F*(2, 94) = 0.74, *p* = .48, *ɳ*_*p*_^*2*^ = .02, see Fig 2), thus there was no group difference in improvement. Because the average overall z-score was close to 0, indicating participants were not severely impaired, we reran the analyses (post-hoc) including only participants who were impaired (i.e., z-score lower than -1.65) on at least one of the outcome measures at baseline, again obtaining similar results as for the primary and secondary analyses mentioned before.

#### Comparison with statistical methods from previous studies

Previous studies typically only performed analyses in the intervention group without direct statistical comparison with a control group or without correcting for multiple testing. To investigate how our results compare to these from previous studies, we also performed within-group analyses (even though we consider these analyses less appropriate, because between-group analyses are required to control for training-unspecific effects). Paired Student t-tests of the 26 outcome measures in the intervention group revealed 10 significant improvements (and 2 trends towards improvement), 3 of which would survive adjustment for multiple comparisons (see Table 4). The waiting list group improved significantly on four outcome measures, none of which would survive multiple comparison adjustment. Thus, without direct statistical comparison of all three groups within the same analyses we would have concluded that brain training results in cognitive improvement.

**Table 4:** Mean (standard deviation) and paired Student's t-test of the difference between before and after study period for all outcome measures in the intervention group and the waiting list group.

	Intervention group (n = 38)						Waiting list group (n = 24)					
Measure	Pre-training		Post-training		*d*^a^	p—value^a^	Pre-waiting		Post-waiting		*d*^a^	p—value^a^
*Primary*												
D-Kefs TMT (switch; z)	0.1	(1.1)	0.2	(1.0)	0.2	.09	-0.3	(1.2)	-0.1	(1.2)	0.2	**.03**
Letter Number Seq. (z)	0.1	(1.1)	0.2	(1.1)	0.1	.26	-0.3	(1.2)	0.0	(1.1)	0.2	.11
Phonetic fluency (z)	-0.4	(1.2)	-0.2	(1.3)	0.1	**.01**	-0.8	(1.0)	-0.8	(0.8)	0.0	1.00
Semantic fluency (words)	20.1	(6.0)	19.9	(6.4)	0.0	.81	17.6	(3.7)	18.3	(4.2)	0.2	.29
ToL (optimal—moves)	-33.4	(22.9)	-25.6	(15.6)	0.4	**.02**	-34.2	(19.8)	-27.6	(14.4)	0.3	.24
*Secondary*												
Cognitive Flexibility												
Fluency Switch (words)	-3.5	(4.2)	-3.3	(4.1)	0.0	.82	-1.6	(2.7)	-2.5	(3.1)	-0.3	.15
Switch RT (ms)	-407	(245)	-357	(222)	0.2	.07	-433	(228)	-461	(241)	-0.1	.44
Switch Acc (trials)	-3.0	(4.0)	-3.0	(3.5)	0.0	1.00	-3.3	(4.0)	-4.3	(4.1)	-0.3	.22
Dual RT (ms)^c^	-390	(368)	-451	(292)	-0.2	.11	-334	(263)	-426	(239)	-0.4	**.02**
Dual Acc (trials)^c^	1.2	(7.6)	0.5	(7.3)	-0.1	.31	-1.3	(4.6)	-0.5	(5.0)	0.1	.29
TMT B online (sec)^b^	85.3	(39.4)	75.5	(31.5)	0.3	**.01**	98.1	(48.9)	87.6	(45.5)	0.3	**.05**
Attention												
TMT A online (sec)^b^	47.8	(21.8)	41.6	(15.7)	0.3	**.01**	52.7	(23.5)	49.3	(23.6)	0.2	.10
PASAT (%)	73.4	(17.3)	76.2	(18.1)	0.2	**.05**	63.2	(22.5)	67.4	(22.4)	0.3	**.03**
DSC online (correct)	35.6	(8.4)	37.9	(8.7)	0.3	< .001*	34.7	(6.5)	35.2	(7.9)	0.1	.40
DSC paper (z)	-0.3	(1.2)	-0.1	(1.2)	0.1	.06	-0.6	(1.0)	-0.5	(1.0)	0.1	.43
Verbal memory												
RAVLT direct (z)	-0.2	(1.4)	0.0	(1.3)	0.1	.25	-0.3	(1.5)	-0.2	(1.4)	0.1	.65
RAVLT delayed (z)	-0.2	(1.1)	-0.3	(1.0)	-0.1	.69	-0.1	(1.3)	0.1	(1.2)	0.1	.67
Working memory												
Corsi (span)	7.1	(1.2)	7.0	(1.1)	-0.1	.58	7.0	(1.2)	6.8	(1.4)	-0.1	.61
N-back (%: 2back-0back)	-15.5	(12.0)	-13.3	(9.9)	0.2	.11	-13.9	(10.1)	-12.7	(8.7)	0.1	.60
Reasoning												
Raven PM (correct)	17.4	(2.2)	17.4	(2.2)	0.0	1.00	17.0	(2.7)	17.1	(2.6)	0.1	.82
Shipley (correct)	14.7	(2.9)	14.9	(3.2)	0.4	**.02**	14.8	(3.7)	14.8	(3.2)	0.3	**< .01**
Psychomotor speed												
Click (sec)^b^	31.5	(11.0)	29.1	(14.0)	0.3	**.01**	44.8	(42.3)	38.6	(33.9)	0.1	.65
Drag (sec)^b^	3.1	(1.8)	2.4	(1.1)	0.5	< .01*	2.7	(1.3)	2.4	(0.9)	0.3	.17
Peg (sec)^b^	73.9	(24.7)	66.7	(20.8)	0.4	< .01*	78.3	(21.1)	75.0	(22.2)	0.2	.24
D-Kefs TMT (motor; z)	0.3	(0.7)	0.3	(0.6)	0.0	.70	0.2	(0.7)	0.2	(0.7)	0.1	.55
Inhibition												
Stop (ms)	-298	(43)	-285	(64)	0.2	.13	-289	(81)	-277	(56)	0.2	.38

Note. These statistics are only to compare with previous studies and are not based on analyses that we consider appropriate. Bold values are considered significant.

* = remains significant after Bonferroni-Holm adjustment

d = Cohen's d (effect size of the difference score, positive values represent improvements)

^a^ = based on the transformed values if variables were transformed for statistical analyses

^b^ = lower values represent better performance

^c^ = before data analyses this tasks was seen as unsuitable for univariate analyses and should only be used in combination with the switch task within a composite score

D-Kefs = Delis-Kaplan Executive Function System; TMT = Trail Making Test; Seq. = Sequencing; ToL = Tower of London; Acc = accuracy; PASAT = Paced Auditory Serial Addition Task; DSST = Digit-Symbol-Coding; PM = Progressive Matrices.

However, as mentioned above, based on repeated-measures MANOVAs when the three groups are compared directly, these group differences are not significant. Lack of statistical power is an unlikely explanation for the absence of group differences, because univariate ANOVAs did also not show significant differences between the three groups (results not reported but available upon request), not even when both training groups were pooled and compared with the waiting list group. Results from Bayesian analyses were also in favor of the H0 (i.e., evidence that the data was more likely to originate from one group, see S1 Table) for the outcome measures that showed no group differences based on the ANOVAs.

#### Per-protocol analyses

The main repeated-measures MANOVAs were repeated for participants who completed the training according to the protocol. Compared to the intention-to-treat analyses, 20 participants were excluded (18 drop-outs, 2 completed < 50 training sessions). Thus, analyses were based on 77 participants (*n*_intervention_ = 28, *n*_active control_ = 29, *n*_waiting list_ = 20). At baseline (T0), participants who did not follow the protocol had significantly lower memory scores (*t*(95) = 2.70, *p* < .01), D-Kefs TMT scores (*t*(23.7) = 2.21, *p* = .04), and there was a trend for lower attention scores (*t*(23.7) = 1.88, *p* = .06) than participants who followed the protocol. They did not differ significantly on the other baseline variables nor on the rehabilitation received during the study.

Results of the training did not differ from the intention-to-treat analyses. Participants improved on training tasks, but there were no transfer effects of the training to the primary outcomes (*F*(10,136) = 0.70, *p* = .73, *ɳ*_*p*_^*2*^ = .05) nor to the secondary outcomes (*F*(14,132) = 0.73, *p* = .74, *ɳ*_*p*_^*2*^ = .07).

#### Follow-up

As there was no training effect, T1 was not evaluated. Results for the training groups at follow-up were analyzed with a repeated-measures MANOVA with T0 and T3 as time-points, followed by the same analysis with T2 and T3 as time-points. Long-term training effects could not be compared to a passive control group, because the waiting list group did not perform T3 after waiting.

From the 10 outcome measures that were repeated 4 weeks after training completion, 8 showed a significant increase from baseline (T0) to follow-up (T3; *F*(10, 62) = 9.86, *p* < .001, *ɳ*_*p*_^*2*^ = .61). Improvements were seen on the TMT A and B, the three mouse tasks, DSST, ToL, and one of the two switch task outcomes (see S2 Table). However, there was no group*time interaction (*F*(10, 62) = 0.59, *p* = .82, *ɳ*_*p*_^*2*^ = .09); thus, both groups increased equally over time. The time effects disappeared after correcting for education, age and time since stroke (*F*(10, 59) = 0.74, *p* =. 69, *ɳ*_*p*_^*2*^ = .11).

Between T2 and T3, only TMT B and one of the two switch task outcomes improved significantly (*p* < .01, *ɳ*_*p*_^*2*^ = .14 and *p* = .04, *ɳ*_*p*_^*2*^ = .06, respectively) and one of the mouse tasks reached significance (*p* = .06, *ɳ*_*p*_^*2*^ = .05). The rest of the tasks scores remained stable between T2 and T3. Again, there were no group differences (*F*(10, 62) = 1.67, *p* = .11, *ɳ*_*p*_^*2*^ = .21).

## Discussion

The aim of the current study was to evaluate whether a commercially available computer-based brain training improves executive functioning after stroke. We included training elements that had been reported in previous studies to enhance the effects of training. With this training program, we found that participants improved on training tasks and on several outcome tasks, with improvements persisting even 4 weeks after training completion. However, when groups were compared, the intervention group did not improve significantly more than the active and waiting list control groups. This implies that the transfer effects of training were small and may be explained largely by variables unrelated to the training, such as the Hawthorne effect.

Our second aim was to evaluate whether studies without proper control groups can draw conclusions about transfer effects. The improvements *over time* we found do corroborate most findings of previous brain training studies for patients with acquired brain injury [8,9,32–39]. Therefore, without comparing the result of the intervention group to those of the waiting list and active control group, we would also have—unjustifiably—concluded that the intervention training was effective. The current study shows that a positive within-group effect (i.e., time effect) does not necessarily imply an effect of training because the time effect may not differ significantly from the one seen in groups that did not train. Our results underscore the importance of including control conditions in the study design and analyses.

For the main executive functioning outcomes, the time effect disappeared after correcting for education. This also replicates previous results [8]. The time effect of the secondary outcome measures was not affected by these corrections. In the current study, education did not correlate strongly with the outcome measures and the effect of each education level differed per outcome measure, which suggests that education resulted in non-systematic variation. It is therefore unlikely that education accounted for the training effect and it is more likely that the time effect disappeared due to covariation with non-systematic, noise-like patterns.

With the same outcome measures as used in the current study, two studies did report transfer effects of training to executive functioning, verbal memory [38], and to attention [34]. Compared with our study, their participants were more severely impaired, suggesting that training programs may be more effective for these patients. A limitation is that an active control group was not included, so their results may be training-unspecific. Another limitation is that only a small number of tasks per cognitive domain was used. It is important, however, to use multiple outcome measures per cognitive domain because an improvement in a small selection of outcome measures taxing the same function is less convincing than improvement on several outcome measures within a function [40]. With the use of composite scores, as we did in our study, only training effects that consistently improve a given domain will be revealed, not merely the skills for one particular task. This would be strong evidence that the training indeed improved the underlying cognitive functioning measured by that set of tasks.

There were several limitations in our study. First, our sample consisted of stroke patients who were relatively high functioning with an average z-score of -0.6. Only 63% of the participants had scores that suggest impairment (z < -1.65) at least in one outcome measure. This may have been due to a selection bias, because participants needed to be able to use a computer and endure the workload of this study. Nevertheless, the training results did not change when we only included these more severely impaired patients.

Second, similar to what was found in two large studies with healthy elderly adults [41,42], the effect size of improvement on outcome measures was small, on average *ɳ*_*p*_^*2*^ = .20 (which corresponds to *d* = 0.29). To reveal these effects with a power of 0.8 and alpha of .05 (one sided), 444 participants (3x 148) would be needed. However, an intervention with such a small effect size is hardly clinically relevant.

Third, the adaptiveness of the training was somewhat compromised in both training groups. As a result, the active control training was more challenging than we had planned, and five intervention group participants were slightly less stimulated because they reached the ceiling in *one of the nine* training tasks during the last phase of their training. This may explain the absence of difference in transfer effects between the intervention and the mock training. However, results without these participants did not differ from those including them, thus rendering the compromised adaptiveness of the training an unlikely candidate to explain the absence of training effects.

Fourth, we expected that training five times per week during half an hour per day for a total of 29 hours would be sufficient to induce restitution-based recovery. Possibly, this still was not sufficient and more time or a higher frequency of training is needed [42,43]. Even though participants were coached during the entire training period and could contact us whenever they ran into problems, they might have benefited from additional face-to-face contact and support [44]. It is also possible that the training was not sufficiently tailored to improve cognitive functioning in patients.

Fifth, a general limitation of executive functioning tasks pertains to learning effects during repeated performance. Yet, no parallel versions were available for the tasks that improved over time. Training effects may thus have been absorbed by these retest effects.

Our study was designed to maximize the likelihood of uncovering training effects on executive functions. Despite our efforts, we did not find any evidence in favor of the notion that computer-based training helps to improve executive functioning. The efforts included the following: Our study had sufficient statistical power to reveal clinically relevant effect sizes. The training included important aspects of a training program that are thought to be essential, and used professionally programmed tasks. These were stimulating and tapped into three cognitive domains. The gaming platform was tailored to our research and allowed control over many aspects of the training, including (forced) task order. The training included rapid task switches and was spread over a long period with a high frequency per week. Participants were coached and motivated to train. Finally, our replication of time effects found by previous studies, and all time effects showing improvements rather than deterioration, indicate that the lack of training transfer is not due to measurement errors.

We recommend that future studies adjust their training to the specific needs of each patient and provide more face-to-face coaching, especially at the beginning of the training. It is important to control essential training elements (e.g., level selection should be done by the program and not by the participant) in order to ensure compliance to study protocol. Finally, studies should include proper control conditions, multiple outcome measures per cognitive domain, and large sample sizes.

Our study showed that a high-potential cognitive flexibility training did not convincingly result in improvements on several cognitive outcome measures. We did find improvements over time, but improvements were similar in all three groups, including in the waiting list group. The lack of training results in this study does not imply that computer training programs in general are ineffective. However, caution is needed when interpreting positive results of previous training studies, because most of them did not include adequate control groups. Based on our results, we cannot recommend general brain training programs that are now commercially available when performed five times per week during three months. A training protocol as used in our study is unlikely to yield clinically relevant benefits for the rehabilitation of cognitive impairments after stroke. It remains possible, however, that training programs with a much higher training dose, or programs tailored to the specific needs of individual patients, will be effective and clinically relevant.

## Supporting information

S1 File Details of data preparation. (PDF)

S1 Fig Improvement on training tasks of the intervention group (n = 36) and the active control group (n = 33). Scores are the average of all training tasks performed (max is 2000, for active control group max should be 900). Error bars represent standard errors. The lines are offset horizontally to reveal both lines.
(PDF)

S1 Table Results from Bayesian independent samples t-test with training groups combined versus waiting list group. (PDF)

S2 Table Mean (standard deviation) and repeated-measures MANOVA of the outcome measures at follow-up. (PDF)

S2 File Protocol approved by Medical Ethical committee. (PDF)

S3 File CONSORT 2010 checklist_TAPASS. (PDF)

S4 File TAPASS_minimal SPSS dataset without demographical information_CVA. (SAV)

## Abbreviations

ANOVA: Analysis of variance
D-Kefs: Delis-Kaplan Executive Function System
DSC: Digit-Symbol-Coding
LNS: Letter Number Sequencing
MANOVA: Multivariate analysis of variance
PASAT: Paced Auditory Serial Addition Task
RAVLT: Rey’s auditory verbal learning test
RCT: Randomized controlled trial
RT: Reaction time
TAPASS: Training Project Amsterdam Seniors and Stroke
TICS: Telephone Interview for Cognitive Status
TMT: Trail Making Test
ToL: Tower of London

## References

[1] Maaijwee NAMM, Schaapsmeerders P, Rutten-Jacobs LCA, Arntz RM, Schoonderwaldt HC, et al (2014) Subjective cognitive failures after stroke in young adults: Prevalent but not related to cognitive impairment. J Neurol 261(7): 1300–1308. 10.1007/s00415-014-7346-3 24740819

[2] Middleton LE, Lam B, Fahmi H, Black SE, McIlroy WE, et al (2014) Frequency of domain-specific cognitive impairment in sub-acute and chronic stroke. Neurorehabilitation 34(2): 305–312. 10.3233/NRE-131030 24401826

[3] Lesniak M, Bak T, Czepiel W, Seniow J, Czlonkowska A. (2008) Frequency and prognostic value of cognitive disorders in stroke patients. Dement Geriatr Cogn Disord 26(4): 356–363. 10.1159/000162262 18852488

[4] Nys GMS, van Zandvoort MJE, van der Worp HB, de Haan EHF, de Kort PLM, et al (2006) Early cognitive impairment predicts long-term depressive symptoms and quality of life after stroke. J Neurol Sci 247(2): 149–156. 10.1016/j.jns.2006.04.005 16716359

[5] Kurland J, Baldwin K, Tauer C. (2010) Treatment-induced neuroplasticity following intensive naming therapy in a case of chronic wernicke's aphasia. Aphasiology 24: 737–751.

[6] Thrane G, Friborg O, Anke A, Indredayik B. (2014) A meta-analysis of constraint-induced movement therapy after stroke. J Rehabil Med 46(9): 833–842. 10.2340/16501977-1859 25182341

[7] van de Ven RM, Murre JMJ, Veltman DJ, Schmand BA. (2016) Computer-based cognitive training for executive functions after stroke: A systematic review. Frontiers in Human Neuroscience 10(150). 10.3389/fnhum.2016.00150 PMC4837156 27148007

[8] Gray JM, Robertson I, Pentland B, Anderson S. (1992) Microcomputer-based attentional retraining after brain damage: A randomised group controlled trial. Neuropsychological Rehabilitation 2(2): 97–115.

[9] Spikman JM, Boelen DHE, Lamberts KF, Brouwer WH, Fasotti L. (2010) Effects of a multifaceted treatment program for executive dysfunction after acquired brain injury on indications of executive functioning in daily life. Journal of the International Neuropsychological Society 16(1): 118–129. 10.1017/S1355617709991020 19900348

[10] Mayo E. (1933) The human problems of an industrial civilization, New York: Macmillan Co.

[11] Roethlisberger FJ, Dickson WJ. (1939) Management and the worker: An account of a research program conducted by the western electric company, Hawthorne works, Chicago Cambridge, Mass.: Harvard University Press.

[12] Glass BD, Maddox WT, Love BC. (2013) Real-time strategy game training: Emergence of a cognitive flexibility trait. PLoS ONE [Electronic Resource] 8(8): e70350. 10.1371/journal.pone.0070350 PMC3737212 23950921

[13] Miyake A, Friedman NP, Emerson MJ, Witzki AH, Howerter A, et al (2000) The unity and diversity of executive functions and their contributions to complex "frontal lobe" tasks: A latent variable analysis. Cognit Psychol 41(1): 49–100. 10.1006/cogp.1999.0734 10945922

[14] Buitenweg JIV, Murre JMJ, Ridderinkhof KR. (2012) Brain training in progress: A review of trainability in healthy seniors. Front Hum Neurosci 6: 183 10.3389/fnhum.2012.00183 22737115 PMC3380254

[15] van de Ven RM, Schmand BA, Groet E, Veltman DJ, Murre JMJ. (2015) The effect of computer-based cognitive flexibility training on recovery of executive function after stroke: Rationale, design and methods of the TAPASS study. BMC Neurology 15(144). 10.1186/s12883-015-0397-y PMC4545547 26286548

[16] Cohen J. (1988) Statistical power analysis for the behavioral sciences Hillsdale, NJ: Lawrence Earlbaum Associates.

[17] Karbach J, Kray J. (2009) How useful is executive control training? Age differences in near and far transfer of task-switching training. Developmental Science 12(6): 978–990. 10.1111/j.1467-7687.2009.00846.x 19840052

[18] Saghaei M, Saghaei S. (2011) Implementation of an open-source customizable minimization program for allocation of patients to parallel groups in clinical trials. Journal of Biomedical Science and Engineering 4: 734–739.

[19] Brandt J, Spencer M, Folstein M. (1988) The telephone interview for cognitive status. Neuropsychiatry Neuropsychol Behav Neurol 1(2): 111–7.

[20] Delis DC, Kaplan E, Kramer J. (2001) Delis–Kaplan executive function system San Antonio, TX: Psychological Corporation.

[21] Thurnstone LL. (1938) Primary mental abilities Chicago: University of Chicago press.

[22] Benton AL, Hamsher K. (1989) Multilingual aphasia examination Iowa City: AJA associates.

[23] Culbertson W, Zillmer E. (2005) Tower of London Drexel University. Chicago: Multi-Health Systems.

[24] Wechsler D. (2000) Wechsler adult intelligence scale (WAIS-III) Nederlandstalige bewerking. Technische handleiding. Lisse: Swets & Zeitlinger.

[25] Rogers RD, Monsell S. (1995) Costs of a predictable switch between simple cognitive tasks. Journal of Experimental Psychology-General 124(2).

[26] Gronwall DMA. (1977) Paced auditory serial-addition task: Measure of recovery from concussion. Percept Mot Skills 44(2): 367–373. 10.2466/pms.1977.44.2.367 866038

[27] Saan R, Deelman B. (1986) De 15-woordentest A en B. (een voorlopige handleiding) Groningen: Afdeling Neuropsychologie, AZG (international publication).

[28] Lever AG, Werkle-Bergner M, Brandmaier AM, Ridderinkhof KR, Geurts HM. (2015) Atypical working memory decline across the adult lifespan in autism spectrum disorder? J Abnorm Psychol 124(4): 1014–1026. 10.1037/abn0000108 26595478

[29] Raven J, Raven JC, Court JH. (1998) Manual for raven's progressive matrices and vocabulary scales—section 3: Standard progressive matrices Oxford: Oxford Psychologists Press.

[30] Zachary RA. (1991) Shipley institute of living scale: Revised manual Los Angeles: Western Psychological Services.

[31] Grubbs FE. (1950) Sample criteria for testing outlying observations. Annals of Mathematical Statistics 21: 27–58.

[32] Lundqvist A, Grundstrom K, Samuelsson K, Ronnberg J. (2010) Computerized training of working memory in a group of patients suffering from acquired brain injury. Brain Injury 24(10): 1173–1183. 10.3109/02699052.2010.498007 20715888

[33] Akerlund E, Esbjornsson E, Sunnerhagen KS, Bjorkdahl A. (2013) Can computerized working memory training improve impaired working memory, cognition and psychological health? Brain Injury 27(13–14): 1649–1657. 10.3109/02699052.2013.830195 24087909

[34] Westerberg H, Jacobaeus H, Hirvikoski T, Clevberger P, Ostensson ML, et al (2007) Computerized working memory training after stroke—A pilot study. Brain Injury 21(1): 21–29. 10.1080/02699050601148726 17364516

[35] van Vleet TM, Chen A, Vernon A, Novakovic-Agopian T, D'Esposito MT. (2015) Tonic and phasic alertness training: A novel treatment for executive control dysfunction following mild traumatic brain injury. Neurocase 21(4): 489–498. 10.1080/13554794.2014.928329 24984231

[36] Ruff RM, Mahaffey R, Engel J, Farrow C, Cox D, et al (1994) Efficacy study of THINKable in the attention and memory retraining of traumatically head-injured patients. Brain Injury 8(1): 3–14. 8124315 10.3109/02699059409150954

[37] Chen SH, Thomas JD, Glueckauf RL, Bracy OL. (1997) The effectiveness of computer-assisted cognitive rehabilitation for persons with traumatic brain injury. Brain Injury 11(3): 197–209. 9058001 10.1080/026990597123647

[38] De Luca R, Calabro RS, Gervasi G, De Salvo S, Bonanno L, et al (2014) Is computer-assisted training effective in improving rehabilitative outcomes after brain injury? A case-control hospital-based study. Disability and Health Journal 7(3): 356–360. 10.1016/j.dhjo.2014.04.003 24947578

[39] Lin Z, Tao J, Gao Y, Yin D, Chen A, et al (2014) Analysis of central mechanism of cognitive training on cognitive impairment after stroke: Resting-state functional magnetic resonance imaging study. J Int Med Res 42(3): 659–668. 10.1177/0300060513505809 24722262

[40] Slagter HA. (2012) Conventional working memory training may not improve intelligence. Trends Cogn Sci (Regul Ed) 16(12): 582–583. 23089361 10.1016/j.tics.2012.10.001

[41] Corbett A, Owen A, Hampshire A, Grahn J, Stenton R, et al (2015) The effect of an online cognitive training package in healthy older adults: An online randomized controlled trial. Journal of the American Medical Directors Association 16(11): 990–997. 10.1016/j.jamda.2015.06.014 26543007

[42] Hardy JL, Nelson RA, Thomason ME, Sternberg DA, Katovich K, et al (2015) Enhancing cognitive abilities with comprehensive training: A large, online, randomized, active-controlled trial. Plos One 10(9): e0134467 10.1371/journal.pone.0134467 26333022 PMC4557999

[43] Bhogal SK, Teasell R, Speechley M. (2003) Intensity of aphasia therapy, impact on recovery. Stroke 34(4): 987–993. 10.1161/01.STR.0000062343.64383.D0 12649521

[44] Lampit A, Hallock H, Valenzuela M. (2014) Computerized cognitive training in cognitively healthy older adults: A systematic review and meta-analysis of effect modifiers. Plos Medicine 11(11): e1001756 10.1371/journal.pmed.1001756 25405755 PMC4236015

