# Supplementary material for: Brain training improves recovery after stroke but waiting list improves equally: A multicenter randomized controlled trial of a computer-based cognitive flexibility training
Source: PLoS One. 2017 Mar 3;12(3):e0172993. doi: 10.1371/journal.pone.0172993 (PMC5336244; doi:10.1371/journal.pone.0172993)
Supplement: S1 File — (PDF) [file pone.0172993.s001.pdf]

Supporting Information 1 – Details of Data Preparation

Primary outcomes

Parallel versions were used for the fluency tasks (i.e., girl names and cities at one time-point and boy names and supermarket articles at the other time-point). The supermarket article and city versions turned out to be unequal. Therefore, the mean difference of both versions at T0 was deducted from the scores on the city category and from the girl city switch category of both T0 and T2.

Originally, we planned to use the D-Kefs TMT number-letter switching condition corrected for the separate letter and number conditions (i.e., condition 2 and 3). While preparing the analyses, we realized that this is not a reliable measure. Crawford [1] reported a reliability of r =-.06. This was confirmed by the test-retest correlation in the waiting-list group (r = -.06). We do not expect that the training would influence the ability to connect numbers or letters and the test-retest design of our study would already correct for these variances. We chose, therefore, to use D-Kefs TMT number-letter switching condition *without* correction for the separate letter and number conditions.

Training results of the D-Kefs TMT, with correction for conditions two and three, did not differ from the main analyses with only age corrected D-Kefs TMT number-letter switching condition. See Table A for the results of the repeated-measures MANCOVA with the D-Kefs TMT corrected for condition 2 and 3 variable.

There was one outlier in category fluency that was replaced and there were two multivariate outliers. Because there was no explanation for the multivariate outliers, they were included in the analyses. In the intention-to-treat analyses, 11.3% of data were imputed via last observation carried forward (or backward) as the participant did not complete the task, for example due to drop-out.

Table A. Mean (standard deviation) and repeated-measures MANOVA of the outcome measures including D-Kefs corrected for condition 2 and 3.

	Group									Comparison					
	Intervention group (n = 38)			Active control group (n = 35)			Waiting list group (n = 24)			Time			Time*group		
measure	Pre-training	Post-training	Δ	Pre-training	Post-training	Δ	Pre-waiting	Post-waiting	Δ	F^{α}	p-value	η_p^2	F^{α}	p-value	η_p^2
primary										$F_{(5, 90)}$	**<.01**	.19	$F_{(10, 182)}$	.93	.02
DKEF TMT (switch)	-0.5 (0.9)	-0.4 (0.9)	0.0	-0.5 (1.1)	-0.4 (0.9)	0.1	-0.6 (0.9)	-0.3 (0.9)	0.4		**.05**	.04			
Letter Number Seq.	0.1 (1.1)	0.2 (1.1)	0.1	0.0 (1.2)	0.2 (1.1)	0.2	-0.3 (1.2)	0.0 (1.1)	0.3		**.01**	.07			
Phonetic fluency	-0.4 (1.2)	-0.2 (1.3)	0.1	-0.7 (1.2)	-0.7 (1.3)	0.1	-0.8 (1.0)	-0.8 (0.8)	0.0		.20	.02			
Semantic fluency	20.1 (6.0)	19.9 (6.4)	-0.1	18.8 (7.4)	18.8 (6.9)	0.0	17.6 (3.7)	18.3 (4.2)	0.7		.61	.00			
Tower of London	-33.4 (22.9)	-25.6 (15.6)	7.8	-34.0 (26.0)	-29.0 (21.5)	4.9	-34.2 (19.8)	-27.6 (14.4)	6.6		**<.01**	.08			

Note. All scores are z-scores except for semantic fluency (words mentoined) and Tower of London (reversed score of extra moves required); Higher scores represent better performance; F was based on Pillai's Trace; η_p^2 = partial eta squared (effect size); DKEF TMT = Delis-Kaplan Executive Function System Trail Making Test condition 4 corrected for condition 2 and 3; Seq. = Sequencing.

Secondary outcomes

Parallel versions were used for the Rey’s auditory verbal learning test (RAVLT) and Raven Progressive Matrices.

Cognitive flexibility was measured by the switch condition of the category fluency task, TMT B, and by a modified version of the switch task [2]. During the switch task, a number and a letter were presented on a computer screen. Every other trial, participants had to switch between either mentioning whether the number was higher or lower than five, and between mentioning whether a letter was uppercase or lowercase. In the dual block, participants had to do the number and letter tasks with each stimulus. They needed to do the first task as fast as possible after which they could take time to do the second task (see [3]for elaborate task descriptions). Trials with a reaction time (RT) below 100 ms, above 5000 ms, or greater than 3 SD above the mean RT for each category of the participant (i.e., switch or no-switch) were removed. Trials following an incorrect trial were also removed. In this way, 20% of trials were removed at T0 and 13% at T2. Switch cost and dual cost were calculated for RT and accuracy (see Table 1 how cost was calculated). The outcome measures in the cognitive flexibility composite score were weighed, because both the dual and the switch cost were represented by two outcome measures (i.e., cognitive flexibility = mean((z TMT B*2), (z fluency switch *2), z switch RT, z switch accuracy, dual RT, dual accuracy).

In the n-back task, participants had to respond whether a picture was a car (0-back) or whether the current picture was the same as two pictures before (2-back). The task consisted of four 0-back and four 2-back blocks. First blocks with a score at or below chance level were removed if the other three blocks were carried out above chance level. In these cases, both the first 0-back and the first 2-back blocks were removed. If the total score of either the 0-back or the 2-back was at or below chance level, the difference percentage score of the n-back was replaced with the worst score of that group at that time-point. In addition, at least half of the trials needed to be present; if not, the difference percentage score was marked as missing.

Similarly, if accuracy of the switch task was at or below chance level, the RT and accuracy cost of both the switch and dual tasks were replaced (as the dual score relies on the no-switch score of the switch task). For the dual task, only the dual RT and accuracy were replaced. In the stop task participants had to indicate the direction of a green arrow but withhold a response whenever the arrow turned red. The cut-off for the chance level for the go trials that were

replaced, was placed at 60% [4]. Participants who had a stop trial accuracy above 90% were marked as missing as they did not adhere to task instruction. For these three tasks, 2.8% of the data was replaced due to chance level scores. The average RT of the drag and drop task needed to be based on at least four of the eight trials otherwise the score was marked as missing.

Outliers were replaced in 0.4% of the secondary outcomes data and 2.3% was marked as missing as the participant was incapable of doing the task or performed at or below chance level. In addition, for the intention-to-treat analyses 11.4% of data were imputed via last observation carried forward (or backward) as the participant did not complete the task, for example due to drop-out.

Several outcome measures were not normally distributed and were thus transformed using the following formulas:

ToL: square root of score

PASAT and Shipley: -1*(square root of ((max score+1)- score))

TMT A, TMT B, and Drag and drop skill: score ^-.14

Drag skill grid and click skill: score^-.4

N-back: score ^2.83

DSST online: score ^1.04

Assumption check for MANCOVA

There appeared to be a significant time since stroke*group interaction for the category fluency task (p = .01). There were also significant time since stroke*cognitive flexibility and time since stroke*reasoning interactions for group. These latter interactions were, however, driven by the multivariate outliers; thus time since stroke was included as covariate and differences in results without these outliers are reported. Results with only education and age as a covariate did not differ from result with time since stroke in addition as a covariate, thus results are reported with time since stroke as a covariate in de the repeated-measures MANCOVAs.

Adjustment for Age, education, and time since stroke

This significant time effect for the primary outcome measures disappeared after correcting for age, time since stroke, and education ($F(5,87) = 0.78$, $p = .57$, $\eta_p^2 = .04$) and the group*time

interaction remained non-significant (F(10,176) = 0.60, p = .81, η_p^2 = .03). Only education significantly accounted for 33% of the variance but the correlation between education and the outcome measures was low (max r = .28) and the effects of education were highly nonsystematic, rendering interpretation of this MANCOVA outcome essentially meaningless.

For the secondary outcome measures, after correcting for age, time since stroke, and education, the significant time effect remained only for attention (p < .01, η_p^2 = .11) and was no longer significant for cognitive flexibility (p = .08, η_p^2 = .03) and psychomotor speed (p = .07, η_p^2 = .04). The group*time interaction remained non-significant (F(14,172) = 0.68, p = .79, η_p^2 = .05).

Waiting list addition

The waiting list group was added to the study design during the course of the study, because inclusion was going smoother than expected before study commencement. Participants who started before the waiting list addition did not differ significantly on baseline and did not have different time or training effects compared to those who started after the waiting list addition.

Technical failures

Technical failures were not often reported (*Mdn* = 2 times per participant, IQ = 0-6) and did not differ between training groups (Mann–Whitney U = 641.0, p = .58). Participants who dropped out did not report more technical failures (*Mdn = .5*) than those who did not drop out (*Mdn* = 2).

Does believe of training effectiveness matter?

Based on explorative analyses, those participants who thought they received the intervention training did not improve significantly more on training tasks than those who thought that they received the mock training (Mann–Whitney U = 341.0, $n_{\text{intervention believe}}$ = 44, $n_{\text{mock believe}}$ = 13, p = .30). Nor did they improve more than those who thought that they received the mock training on primary transfer tasks (F(5,67) = 0.73, p = .60, η_p^2 = .05) and secondary transfer tasks (F(7,65) = 0.53, p = .81, η_p^2 = .05).

References

1. Crawford JR, Sutherland D, Garthwaite PH. (2008) On the reliability and standard errors of measurement of contrast measures from the D-KEFS. Journal of the International Neuropsychological Society 14(6): 1069-1073.

2. Rogers RD, Monsell S. (1995) Costs of a predictable switch between simple cognitive tasks. Journal of Experimental Psychology-General 124(2).

3. van de Ven RM, Schmand BA, Groet E, Veltman DJ, Murre JMJ. (2015) The effect of computer-based cognitive flexibility training on recovery of executive function after stroke: Rationale, design and methods of the TAPASS study. BioMed Central NEUROLOGY 15(144).

4. van Muijden J, Band GPH, Hommel B. (2012) Online games training aging brains: Limited transfer to cognitive control functions. Frontiers in Human Neuroscience 6: 1-13.
