# Supplementary material for: Brain training improves recovery after stroke but waiting list improves equally: A multicenter randomized controlled trial of a computer-based cognitive flexibility training
Source: PLoS One. 2017 Mar 3;12(3):e0172993. doi: 10.1371/journal.pone.0172993 (PMC5336244; doi:10.1371/journal.pone.0172993)
Supplement: S1 Fig — Scores are the average of all training tasks performed (max is 2000, for active control group max should be 900). Error bars represent standard errors. The lines are offset horizontally to reveal both lines. (PDF) [file pone.0172993.s002.pdf]

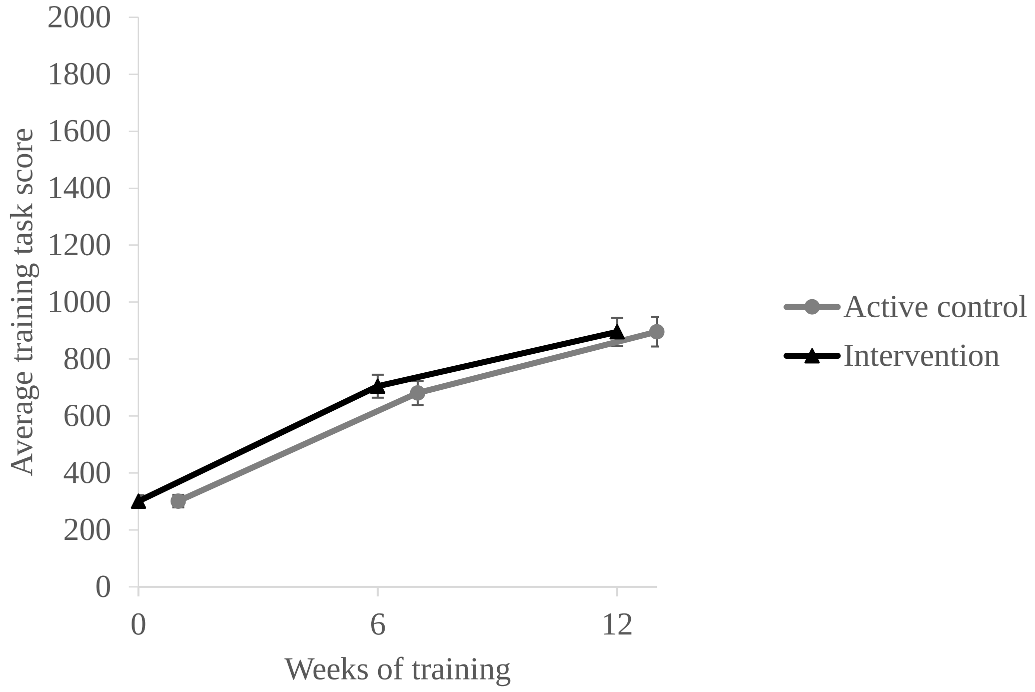

***S1 Fig*. Improvement on training tasks of the intervention group (n = 36) and the active control group (n = 33)**. Scores are the average of all training tasks performed (max is 2000, for active control group max should be 900). Error bars represent standard errors. The lines are offset horizontally to reveal both lines.
