# Supplementary material for: Brain training improves recovery after stroke but waiting list improves equally: A multicenter randomized controlled trial of a computer-based cognitive flexibility training
Source: PLoS One. 2017 Mar 3;12(3):e0172993. doi: 10.1371/journal.pone.0172993 (PMC5336244; doi:10.1371/journal.pone.0172993)
Supplement: S1 Table — (PDF) [file pone.0172993.s003.pdf]

**S2 Table. Results from Bayesian independent samples t-test with training groups combined versus waiting list group.**

| Moderate evidence for H0 | $BF_{01}$ | Anecdotal evidence for H0 | $BF_{01}$ | Evidence for H1 | $BF_{01}$ |
|---|---|---|---|---|---|
| Inhibition | 3.77 | Click | 2.87 | DSC online | 0.82 |
| Corsi | 3.77 | Semantic fluency | 2.52 | | |
| Shipley | 3.75 | Switch Acc | 2.47 | | |
| TMT B | 3.74 | Peg | 2.29 | | |
| Raven PM | 3.70 | Fluency Switch | 2.28 | | |
| ToL | 3.60 | Drag | 2.20 | | |
| N-back | 3.58 | Phonetic fluency | 2.04 | | |
| PASAT | 3.57 | Dual Acc | 1.65 | | |
| DKEF TMT motor | 3.57 | Switch RT | 1.04 | | |
| RAVLT direct | 3.57 | | | | |
| DKEF TMT switch | 3.42 | | | | |
| TMT A | 3.41 | | | | |
| Dual RT | 3.35 | | | | |
| RAVLT delayed | 3.25 | | | | |
| DSC paper | 3.13 | | | | |
| Letter Number Seq. | 3.07 | | | | |

*Note* . Larger values represent more evidence for H0. For example, $BF_{01}$ of 3 indicates that H0 is 3 times more likely than H1. Likewise, values lower than one represent evidence in favor of H1, thus, 0.33 indicates that the H1 is 3 times more likely than H0. H1: training groups ≠ waiting list group. DKEF= Delis-Kaplan Executive Function System; TMT = Trail Making Test; Seq. = Sequencing; ToL = Tower of London; Acc = accuracy; PASAT = Paced Auditory Serial Addition Task; DSST = Digit-Symbol-Coding; PM = Progressive Matrices.
