# Supplementary material for: Brain training improves recovery after stroke but waiting list improves equally: A multicenter randomized controlled trial of a computer-based cognitive flexibility training
Source: PLoS One. 2017 Mar 3;12(3):e0172993. doi: 10.1371/journal.pone.0172993 (PMC5336244; doi:10.1371/journal.pone.0172993)
Supplement: S2 Table — (PDF) [file pone.0172993.s004.pdf]

S3 Table. Mean (standard deviation) and repeated-measures MANOVA of the outcome measures at follow-up.

	Intervention group (n = 38)	Active control group (n = 35)			Comparison											
					Time (T0T3)			Time*group			Time (T2T3)			Time*group		
measure	Follow-up	Pre-training	Post-training	Follow-up	F^{α}	p-value	η_p^2	F^{α}	p-value	η_p^2	F^{α}	p-value	η_p^2	F^{α}	p-value	η_p^2
					$F_{(10, 62)}$	**<.001***	.61	$F_{(10, 62)}$	.82	.09	$F_{(10, 62)}$	**<.001***	.39	$F_{(10, 62)}$	.11	.21
ToL (optimal - moves)	-26.9 (17.8)	-34.0 (26.0)	-29.0 (21.5)	-25.6 (19.4)	9.2	**<.001***	.11				0.7	.41	.01			
Switch RT (ms)	-321 (218)	-418 (328)	-416 (316)	-378 (293)	6.8	**.01***	.09				4.3	**.04**	.06			
Switch Acc (trials)	-3.0 (3.4)	-2.2 (7.3)	-3.0 (4.9)	-3.4 (5.5)	0.8	.36	.01				0.2	.65	.00			
TMT B (sec)[b]	74.8 (37.6)	110.5 (47.8)	102.1 (42.3)	87.8 (41.9)	28.1	**<.001***	.28				11.4	**<.01***	.14			
TMT A (sec)[b]	41.4 (15.1)	57.3 (20.7)	52.5 (24.3)	48.4 (20.2)	33.3	**<.001***	.32				1.8	.18	.03			
DSC online (correct)	38.5 (8.3)	31.7 (7.4)	35.0 (7.8)	34.7 (7.7)	31.1	**<.001***	.30				0.2	.69	.00			
Corsi (span)	7.2 (1.2)	6.8 (1.1)	7.0 (1.0)	6.9 (1.1)	0.4	.52	.01				0.2	.70	.00			
Click (sec)[b]	28.7 (11.8)	37.6 (22.1)	30.9 (17.2)	31.3 (17.1)	12.7	**<.001***	.15				0.1	.73	.00			
Drag (sec)[b]	2.1 (0.7)	3.4 (2.1)	2.7 (1.5)	2.6 (1.3)	29.3	**<.001***	.29				3.8	.06	.05			
Peg (sec)[b]	66.2 (17.1)	83.5 (35.9)	71.0 (21.1)	73.6 (22.0)	20.5	**<.001***	.22				2.9	.09	.04			

Note. Follow-up scores were only available for a slection of the tasks. Bold values are considered significant. Pre- and post-training scores of the intervention group can be found in table 4. * = remains significant after Bonferroni-Holm adjustment; b = lower values represent better performance; T0 = pre-training; T2 = post-training; T3 = 4 weeks after training completion; TMT = Trail Making Test; ToL = Tower of London; Acc = accuracy; DSST = Digit-Symbol-Coding.
