# Supplementary material for: Brain training improves recovery after stroke but waiting list improves equally: A multicenter randomized controlled trial of a computer-based cognitive flexibility training
Source: PLoS One. 2017 Mar 3;12(3):e0172993. doi: 10.1371/journal.pone.0172993 (PMC5336244; doi:10.1371/journal.pone.0172993)
Supplement: S2 File — (PDF) [file pone.0172993.s005.pdf]

Computer based cognitive flexibility training after stroke

Version 1.1: 07-06-2013

Universiteit van Amsterdam

PROTOCOL TITLE: 'Trainings Project Amsterdam Senior and Stroke' (TAPASS study)

**Short title**	**TAPASS study**
**Version**	**1.1**
**Date**	**07-06-2013**
**Principal investigator(s) (in Dutch: hoofdonderzoeker/uitvoerder)**	**prof. dr. D. Veltman**<br>**Professor of Neuro-Imaging in Psychiatric Disorders, Mood and Anxiety Disorders at the VUmc**<br>**d.j.veltman@vumc.nl**
**Research Group**	**prof. dr. J.M.J. Murre**<br>**Professor of Theoretical Neuropsychology at the UvA**<br>**j.m.j.murre@uva.nl**<br><br>**prof. dr. B. Schmand**<br>**Professor of Clinical Neuropsychology at the UvA & Clinical Neuropsychologist at the AMC Neurology**<br>**b.schmand@amc.uva.nl**<br><br>**mw. drs. R.M. van de Ven**<br><br>**PhD-student at the UvA**<br><br>**r.m.vandeven@uva.nl**<br><br>**Prof. dr. K.R. Ridderinkhof**<br>**Professor of Neurocognitive Development and Aging at the UvA**<br>**K.R.Ridderinkhof@uva.nl**<br><br>**mw. drs. J.I.V. Buitenweg**<br>**PhD-student at the UvA**<br>**j.i.v.buitenweg@uva.nl**<br><br>**Prof. dr. F Barkhof**<br>**Professor of Neuroradiology at the VUmc**<br>**f.barkhof@vumc.nl**<br><br>**mw. drs. E. Groet**<br>**Clinical Neuropsychologist & Psychologist at Heliomare**<br>**e.groet@heliomare.nl**<br><br>**mw. dr. S. Kruiper-Doesborgh**<br>**Clinical Psychologist at Reade**<br>**s.kruiper@reade.nl**<br><br>**dr. S.K. Schiemanck**<br>**Rehabilitation Specialist in Neurorehabilitation at AMC**

**Independent physician(s)**	**dr. M.C. Visser**<br>**Neurologist at the VUmc**<br>**mc.visser@vumc.nl**<br><br>**dr. C. van Bennekom**<br>**Rehabilitation Physician & Research Coordinator at Heliomare**<br>**c.vanbennekom@heliomare.nl**
**Sponsor (in Dutch: verrichter/opdrachtgever)**	**Nederlandse Organisatie voor Wetenschappelijk Onderzoek (NWO)**

PROTOCOL SIGNATURE SHEET

Name	Signature	Date
**Head of Department:**<br><br>**Prof. dr. A.T.F. Beekman**<br>**Head of Psychiatry Department**<br>**a.beekman@ggzingeest.nl**	**A.T.F. B**	**06-05-2013**
**Principal Investigator:**<br><br>**prof. dr. D. Veltman**<br>**Professor of Neuro-Imaging in Psychiatric Disorders, Mood and Anxiety Disorders**<br>**d.j.veltman@vumc.nl**	**D.J. V**	**06-05-2013**

Table of Contents

1. INTRODUCTION & RATIONALE 9
2. OBJECTIVE OF TAPASS 9
2.1. Hypotheses 9
3. STUDY DESIGN 10
4. STUDY SAMPLE 11
4.1. Sub-studies 12
4.2. Inclusion criteria 12
4.3. Exclusion criteria 12
4.4. Sample size calculation 13
5. TREATMENT OF SUBJECTS 14
5.1. Intervention 14
6. METHODS 15
6.1. Study parameters 15
6.1.1 Primary outcome parameters 16
6.1.2. Secondary outcome parameters 16
6.1.3. Other parameters 18
6.2. Randomisation, blinding, and treatment allocation 19
6.3. Study procedure 19
6.4. Withdrawal of individual subjects 22
6.5. Replacement of individual subjects after withdrawal 22
6.6. Follow-up of subjects withdrawn from treatment 22
6.7. Premature termination of the study 22
7. SAFETY REPORTING 22
7.1. Section 10 WMO event 22
7.2. Adverse and serious adverse events 22
7.3. Follow-up of adverse events 23
8. STATISTICAL ANALYSIS 23
8.1. Descriptive statistics 23
8.2. Primary and secondary analysis 23
9. ETHICAL CONSIDERATIONS 24
9.1 Regulation statement 24
9.2. Recruitment and consent 24
9.3. Benefits and risks assessment, group relatedness 25
9.4. Compensation for injury 26

9.5. Incentives 26
10. ADMINISTRATIVE ASPECTS AND PUBLICATION 26
10.1 Handling and storage of data and documents 26
10.2 Amendments 26
10.3. Annual progress report 26
10.4. End of study report 27
10.5. Public disclosure and publication policy 27
Appendix 1. MRI screening form 31
Appendix 2. Description of training tasks 32
Appendix 3. Measures at different time points 37
Appendix 4. Description and duration of measures 39
Appendix 5. Composite scores of several cognitive domains 40
Appendix 6. Scan protocol 41

LIST OF ABBREVIATIONS AND RELEVANT DEFINITIONS

CFQ	Cognitive Failure Questionnaire
CIS-F	Checklist Individual Strength- Fatigue subscale
CPM	Raven Coloured Progressive Matrices
DBC	Diagnose Behandeling Combinatie
DEX	dysexecutive questionnaire
D-Kefs	Delis- Kaplan Executive Function System
DSST	Digit Symbol substitution Task
DTI	Diffusion Tensor Imaging
fMRI	Functional Magnetic Resonance imaging
GIT-II	Groninger Intelligentie Test -2
HADS	Hospital Anxiety Depression Scale
IADL	Instrumental activity of daily life scale
IMP	Investigational Medicinal Product
METC	Medical research ethics committee (MREC); in Dutch: medisch ethische toetsing commissie (METC)
MRI	Magnetic Resonance Imaging
O-span	Operation-span
PASAT	Pased Auditory Serial Addition Test
RAVLT	Rey Auditory Verbal Learning Task
SAE	Serious Adverse Event
SF-36	Short Form Health Survey
TICS	Telephone Interview Cognitive Status
TMT	Trail Making Test
ToL	Tower of London
USER-P	Utrechtse Schaal voor Evaluatie en Revalidatie- Participatie
UvA	University of Amsterdam
VBM	Voxel Based Morphometry
WAIS	Wechsler adult intelligence scale
WGMO).	Wet op de geneeskundige behandelovereenkomst
WMO	Medical Research Involving Human Subjects Act (in Dutch: Wet Medisch-wetenschappelijk Onderzoek met Mensen

SUMMARY

Rationale: There is a great need for cognitive rehabilitation in stroke patients. One promising way to ameliorate cognitive impairments after stroke is to use computer games as cognitive exercises. A recent review by our group indicates that “brain training” in elderly is beneficial if the training emphasizes cognitive flexibility. From this we hypothesize that it is highly likely that cognitive flexibility training will also result in cognitive improvements in stroke patients. Furthermore, in several studies, changes in brain activity have been observed after intensive cognitive training. Against this background, we planned the ‘Training Project Amsterdam Seniors and Stroke’ (TAPASS) study

Objective: In this study the effectiveness of online cognitive flexibility training on cognitive functioning in stroke patients will be investigated.

The main research questions are:

Does online cognitive flexibility training result in improved executive functioning in stroke survivors?

How are the changes in cognitive functioning related to neuronal alterations?

Firstly, we expect that online cognitive flexibility training will improve executive functioning in stroke patients. Secondly, we expect that cognitive improvement will be related to changes in brain activity.

Study design: multicenter, double blind, randomized, controlled intervention study with active control group.

Study population: Stroke patients either 3-6 months post-stroke (n = 80) or 12-36 months post stroke (n = 80), who receive or have received cognitive rehabilitation and currently still have cognitive complaints. Patients with severe medical diseases other than stroke, acute psychiatric disorders, severe aphasia or neglect, or inability to use computers are excluded from the study.

Intervention: Participants will be randomized over 12 weeks of online cognitive flexibility training or online mock training (active control).

Main study parameters/endpoints: These patient groups will be compared at baseline, after six weeks and twelve weeks of training, and four weeks after the end of the training. Furthermore, they will be compared to healthy elderly who had the same training. The primary study endpoint is objective executive function. Secondary measures are improvement on training tasks, cognitive flexibility, objective cognitive functioning in other domains than the executive domain, subjective cognitive and everyday life functioning, and neuronal correlates assessed by Magnetic Resonance Imaging. Sociodemographic and clinical data, training compliance, pre-training computer experience and rehabilitation therapy will be included as control measures.

Nature and extent of the burden and risks associated with participation, benefit and group relatedness: All participants will have to train at home during at least 30 minutes per day for five days per week for 12 weeks. Furthermore, participants will be assessed several times. In total, including training, participants will spend 50 hours on this study. We expect that participants will not find the training burdensome because it is designed to be interesting and challenging. Participants who participate in the MRI part of the study will be scanned twice for one hour. To our knowledge there are no risks involved. Difference in benefit is expected between the training and the active control condition. So far none of the treatments has been proven to be superior to the other**.** Moreover, both groups will be offered the opportunity to train with the tasks used in the intervention group after the study period.

It is expected that intensive computer based cognitive flexibility training will result in improvement of executive functioning. Patients may benefit from this in their everyday life activities. Furthermore, results of this study can be helpful for development of treatments that assist the recovery of people suffering from stroke. Risks of computer-based training are low. These potential benefits and the limited risks justify the implementation of the study.

1. INTRODUCTION & RATIONALE

There is a great need for cognitive rehabilitation in stroke patients. More than half of the stroke patients suffer from cognitive impairment three months post-stroke (Madureira, Guerreiro, & Ferro, 2001). Even in the chronic phase, approximately 35% of stroke survivors still suffer from cognitive impairment (Tatemichi et al., 1994). One promising way to ameliorate these impairments is to use computer games as cognitive exercises. Some studies have been unable to find convincing proof for the effects of 'brain training games' (Owen et al., 2010). In a recent review by our group, however, we concluded that cognitive training in healthy elderly subjects may result in cognitive improvement, provided that it includes frequent switching between various training tasks (Buitenweg, Murre, & Ridderinkhof, 2012). Such cognitive flexibility training improved cognitive functioning even in tasks that were not the focus of training, i.e. the effects of the training generalized to so called 'far transfer tasks' (Karbach & Kray, 2009).

Transfer of training, especially generalization of training effects to the patient's daily life, is essential for clinical application of any rehabilitation technique. Moreover, cognitive training results are commonly best in those people who start at a lower functional performance level (Johansson & Tornmalm, 2012; Peretz et al., 2011). Thus, it is highly likely that cognitive flexibility training will result in cognitive improvements in stroke patients who suffer from cognitive impairments. Furthermore, in several studies, even if behavioral training effects were small, changes in brain activity have been observed after intensive cognitive training (e.g.Belleville et al., 2011; Dahlin, Neely, Larsson, Backman, & Nyberg, 2008; Mozolic, Hayasaka, & Laurienti, 2010). Against this background we planned the 'Training Project Amsterdam Seniors and Stroke' (TAPASS) study.

2. OBJECTIVE OF TAPASS

General: To determine whether cognitive flexibility training can improve executive functioning in stroke patients and to investigate how changes in executive functioning are related to neuronal alterations.

Specific: To examine whether cognitive flexibility training improves objective executive functioning after stroke over and above improvements due to care as usual and spontaneous recovery. More so, we want to determine whether cognitive flexibility training results in better executive improvement compared to an active control training. A further objective is to determine the neuronal correlates of executive improvements after cognitive flexibility training. To this end, resting state fMRI and Diffusion Tensor Imaging (DTI) scans will be made in part of the sample. Finally, we will study whether cognitive flexibility training is more beneficial for those with lower baseline executive performance (patients compared to healthy adults) and whether this training is more beneficial in the post-acute or the chronic phase post-stroke.

2.1. Hypotheses

Main: We expect that cognitive flexibility training will result in a larger improvement in objective executive functioning compared with those who receive mock training.

Secondary: It is predicted that cognitive flexibility training will be more effective in stroke patients compared with healthy adults and more effective in the post-acute phase than in the chronic phase post-stroke. Moreover, we expect that cognitive improvement will be related to changes in brain activity. In particular, we expect that resting-state brain activity of stroke patients who receive cognitive flexibility training will converge more to "normal" than of those who did not receive this training.

Explorative: We will explore which lesion characteristics (e.g. type of stroke, size of lesion, brain regions), and other variables (e.g. IQ, age, comorbidities, cognitive flexibility at baseline) predict good outcome.

3. STUDY DESIGN

Multicenter, double blind, randomized, controlled intervention study with active control group. Imaging will be performed at a single location. A schematic overview of the study design can be found in figure 1.

Figure 1: Study design flowchart

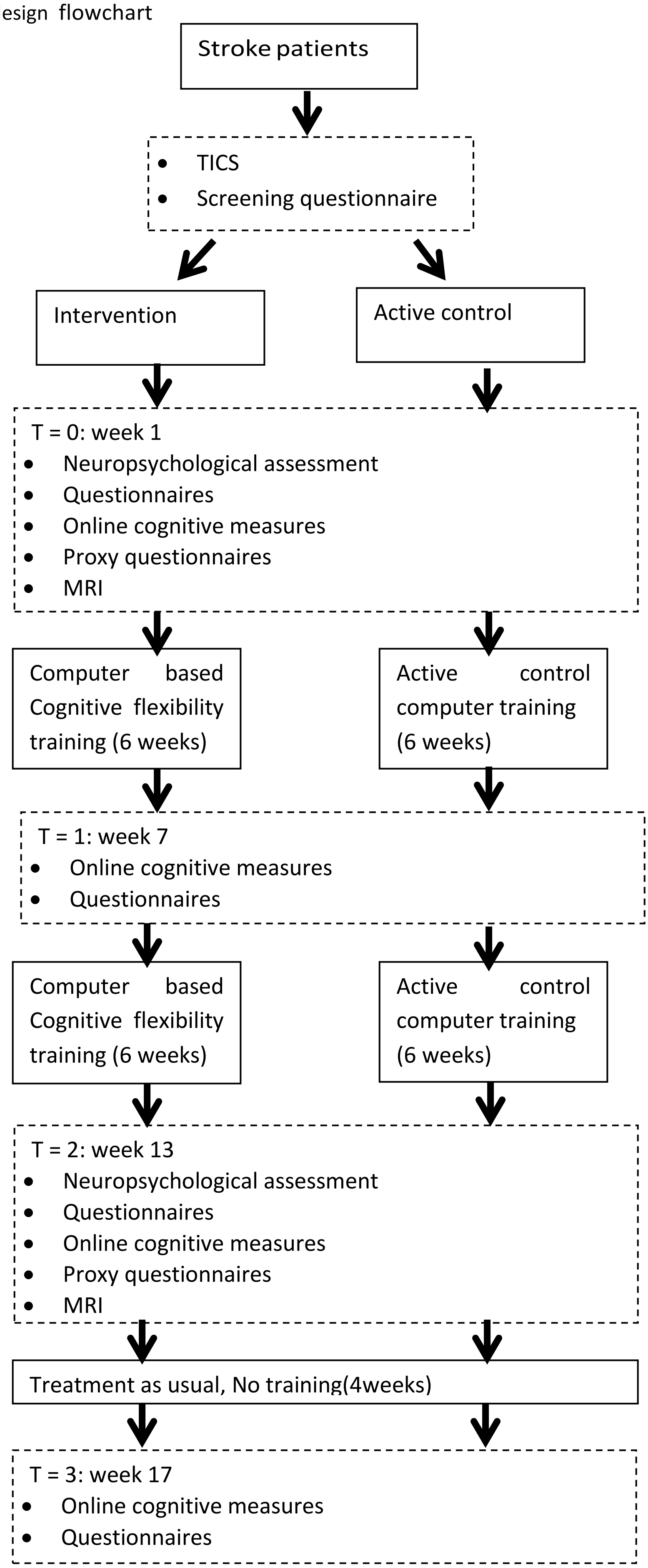

4. STUDY SAMPLE

4.1. Sub-studies

The study is subdivided into two sub-studies. The two studies will include patients who had a stroke and are now between 30 and 80 years old and received rehabilitation as inpatient or outpatient. These patients will be assigned to either 1) a post-acute study: patients who are three to six months post-stroke; or 2) a chronic study: patients who are one to five years post-stroke and who received rehabilitation immediately after stroke. Both patient groups may still receive outpatient rehabilitation treatment and will mostly be living at home again.

The results of these studies will be compared with a third study which will look at healthy adults (n= 120) in the age range of 65 to 80 years. Details of this latter study, which will have the same methodology as discussed below, will be explained elsewhere and is not part of the present protocol.

In each sub-study, part of the sample (those who are able to come to the UvA and fulfill all inclusion and exclusion criteria) will be invited to have two resting state fMRI scans, one at baseline and the second after the training phase. For the fMRI part of the study we aim to include only subjects with middle cerebral artery infarctions.

Sample size: - post-acute stroke patients n=80 (20 per group will be scanned with MRI).

- chronic stroke patients n= 80 (20 per group will be scanned with MRI)

4.2. Inclusion criteria

All patients suffered from stroke and were referred to cognitive rehabilitation. Patients had cognitive dysfunction (not merely subjective complaints), as demonstrated by a neuropsychological assessment or as judged by a neurologist, physiatrist, psychologist, or other experienced clinician. At study entry patients must still have cognitive complaints. Finally, participants must have daily access to a computer with internet connection (compatible with html5) and sound (either through headset or speakers), must be able to independently send emails (e.g. open emails and click on links), and be able to smoothly use the mouse.

4.3. Exclusion criteria

Neurodegenerative disease; epilepsy; serious psychiatric illness (e.g. history of multiple psychotic episodes, acute psychosis, acute major depression); any disease other than stroke which results in severe cognitive impairments; drug or alcohol dependency; severe color blindness; severe aphasia; severe neglect; invalidating vision or hearing problems; severe computer fear disabling the participants to fully complete the neuropsychological assessment and training; and diagnosed learning disability (i.e. mental retardation);. Furthermore, patients who are not mentally fit (defined by Telephone Interview Cognitive Status (TICS) score < 26) and who are not physically fit enough (e.g. medically unstable) to be able to complete 12 weeks of training will be excluded. Finally, those who are not able to understand the training instructions or who cannot execute training due to any other unforeseen

reason, after instructions or after the first training week, will be excluded. New participants will be recruited to replace them.

For the MRI part of the study additional exclusion criteria are contraindications to MRI (see appendix 1), such as presence of metal parts in the body and claustrophobia, and unable to walk a small distance.

4.4. Sample size calculation

We are interested in large differences between the treatment and active control groups. A difference of one standard deviation is considered clinically significant. Therefore, based on a power of .80 and alpha of .05 (one tailed), an effect size of 1 standard deviation will be detected with a sample size of 28 (2x14). A large effect size (d= .80) will be detected at a sample size of 40 (2x 20).

Study 1 (post-acute phase after stroke): Patients from two rehabilitation centers (Reade and Heliomare) are recruited for this study. These rehabilitation centers have approximately 250 stroke patients per year of whom at least 35% will still have cognitive complaints and approximately 2/3 will also fulfill other study requirements. Therefore, in one year about 58 patients (23,3% of total sample) per center will fulfill requirements for participation in our study. We expect that 35% of these patients will participate in our study. Thus, per year and per center it is expected that approximately 20 post-acute stroke patients will be recruited and randomly assigned to one of two training conditions. In case of insufficient inclusion in the two above mentioned centers we will recruit from other additional centers.

When including 80 (2*40) participants and with a power of .80 and alpha of .05 (one- tailed) an effect size of .56 (medium) can be detected. Karbach and Kray (2009) found an average effect size for far transfer effects of .40. However, this effect size was already found after four training sessions. Therefore, it is expected that in our training, with 60 sessions, the effect size will be at least .56. Furthermore, Westerberg et al. (2007) found an effect size of .80 for subjective cognitive complaints (measured by the CFQ) after 16.6 hours of working memory training (Westerberg et al., 2007). This effect would be already revealed with the inclusion of 40 participants.

Study 2 (chronic phase after stroke): Similar to study 1, chronic stroke patients will be recruited from the same rehabilitation centers. Again resulting in approximately 54 patients per center per year who would fulfill inclusion and exclusion criteria. However, it is expected that only 10% of these patients in the chronic phase will participate in our study; resulting in 5 to 6 patients per year. This study will include patients who had a stroke 1 to 3 years ago. Thus, with five centers a total of 75 (5*5*3) are expected to participate in this study. Furthermore, patients will also be recruited by advertisements in patient society newsletters. It is expected that this will result in an additional 5-10 participants. We include 80 participants (2x 40) with whom an effect size of .56 (medium to large) can be detected. An overview of sample size per study can be found in table 1.

MRI: In both studies, a subset of participants will be scanned. So far it is not known what the effect size will be of the MRI outcome measures. Earlier studies that were able to reveal neural changes related to training included 11- 33 participants per condition (Belleville et al., 2011; Dahlin, Neely, Larsson, Backman, & Nyberg, 2008; Mozolic, Hayasaka, & Laurienti, 2010). However, these studies have been performed in healthy subjects who probably have less variability in brain anatomy. It is nevertheless expected that a sample size of 20 per condition should be sufficient to demonstrate neural alterations related to intensive cognitive training. At follow-up, due to potential dropouts we expect to reexamine 65% of participant scanned at T0. Therefore, 13 participants per condition are expected to be analyzed which should be sufficient for the planned analyzes.

Table 1. Sample size: Number of participants in each group (number of MRI participants)

Study	Cognitive Flexibility training	Active controls (mock training)
Post-acute (study 1)	40 (20)	40 (20)
Chronic (study 2)	40 (20)	40 (20)

5. TREATMENT OF SUBJECTS

5.1. Intervention

There is one intervention group (cognitive flexibility training) and an active control group (mock training). Both groups will receive 30 minutes of training per day, with tasks which will be presented online (www.braingymmer.nl). The tasks are designed to be visually attractive and motivating. Feedback will be provided based on personal scores. After each task, feedback will be given visually on a three star rating scale. More stars will be given with higher performance. At the end of each training session participants will be provided with more detailed feedback of their scores on each task.

The intervention group will receive cognitive flexibility training with nine tasks. The tasks selected are intended to train three cognitive domains: attention, reasoning, and working memory. A more elaborate description of these tasks can be found in appendix 2. The cognitive flexibility training will provide several tasks within one session and participants will be asked to frequently switch between these tasks. Each day they will train on 12 tasks for approximately two to three minutes each. Tasks are presented directly after each other to assure that cognitive flexibility, i.e. switching from on task to the other, is required. However, they will train for 15 minutes per task in the first week. Therefore, participants will train on three tasks per day in the first week. This is done to assure that the participants can master all tasks. From the second week on, the number of trials per task will be reduced to assure frequent switching between tasks.

The difficulty of the task (i.e. difficulty level presented to the patient) will be based on the score of the participant. Per day a training session (i.e. workout) with the tasks of that day will be set up for each participant.

The order of the tasks assures that tasks within one cognitive domain (attention, reasoning, and working memory) are not presented directly after each other.

The active control group receives a mock training consisting of four computer tasks which in our view are not likely to improve executive functioning (see appendix 2 for more elaborate descriptions of the tasks). The control tasks will be offered in the same online environment as the intervention (Braingymmer). Five levels of the tasks will be selected and presented to the control group. These levels will be selected to be challenging enough but not too difficult. The active control group will train at one of these levels per week and the level of that week will be randomly chosen. The control training is designed to equalize amount of feedback, visual stimulation, and use of mouse with the intervention training. The active control group will switch tasks approximately every 10 minutes, thus playing three tasks per day.

Both cognitive training and the control (mock) training will take 12 weeks, in which participants will train five times per week for 30- 45 minutes. Furthermore, the active control group will receive the same amount of personal attention (e.g. phone calls) and motivational instructions as the intervention group (see procedure section).

6. METHODS

6.1. Study parameters

Measurement

Prior to training (T=0) and at the end of the training period (T=2) several neuropsychological tasks and computer skill tasks will be administered to measure near transfer (i.e. tasks that are similar to training tasks) and far transfer effects of the training (see appendix 3). Furthermore, several questionnaires will be administered at these time points to determine health status, participation in daily life activities, and subjective cognitive functioning. Some of the measurements are performed online (see appendix 3). In order to compare our study with recent training studies which mostly consisted of 6 weeks of training or shorter (e.g., Lundqvist, Grundstrom, Samuelsson, Ronnberg, 2010; Prokopenko et al., 2013; Westerberg et al., 2007) a subset of online tasks and questionnaires will be administered 6 weeks after training onset (T=1; see appendix 3). Finally, to measure long-term effects of the training a subset of online tasks and questionnaires will be administered 4 weeks after training completion (T=3; see appendix 3).

During training, subjective performance on the training tasks, motivation and fatigue during training, and physical activity at the day of the training will be registered by the participant in an online daily log which will take less than five minutes to fill in. After six and 12 weeks of training, a small questionnaire regarding treatment success will be administered. The primary outcome of this study is objective executive functioning. A global outline of all measurement time-points can be found in table 2. The duration of the assessment sessions can be found in appendix 3. The administration duration of neuropsychological and computer measures can be found in appendix 4.

Table 2. Global outline of measurements per time-point. Note: a more specific outline can be found in appendix 3.

	T0	T1	T2	T3
Neuropsychological assessment	X		X	
Questionnaires	X	X	X	X
Online cognitive measures	X	X	X	X
Proxy questionnaires	X		X	
MRI	X		X	

Study parameters

6.1.1 Primary outcome parameters

An executive function composite score will be calculated based on: D-Kefs TMT letter-number sequencing condition corrected for the separate letter and number conditions (Delis, Kaplan, & Kramer, 2001); Category fluency (GIT-II; Luteijn & Barelds, 2004); Letter fluency (Schmand, Groenink, & van de Dungen, 2008); Tower of London* (ToL; Culbertson & Zillmer, 2005); and Wechsler adult intelligence scale Letter-number sequencing (WAIS III-NL; Wechsler, 2000). These tasks will take in total 30 minutes and will be administered twice (immediately before and immediately after the training phase; T0 and T2).

* = Online measures

6.1.2. Secondary outcome parameters

Training related measures, in particular cognitive flexibility as measured by switch cost and dual task cost are derived from the dual / switching task which is a combined version of a switch task (Rogers & Monsell, 1995) and a modified dual task (Stablum, Umilta, Mazzoldi, Pastore, & Magon, 2007). These tasks will take 30 minutes and will be administered online four times (T0, T1, T2, T3). Furthermore, improvement on training tasks will be registered automatically by the training website.

<u>Objective cognitive functioning</u>

Twelve neuropsychological and computer tasks (see appendix 5) are administered which in total will take 145 minutes. In appendix 3 it can be found when these measures are administered. The obtained scores will be transformed into demographically corrected standard scores. To reduce number of dependent variables, composite scores will be calculated of the following cognitive domains: attention, memory, working memory, intelligence, psychomotor speed.

<u>Subjective functioning</u>

Five questionnaires will be administered to assess subjective dysfunction: subjective executive dysfunction: dysexecutive questionnaire (DEX)* (participant and proxy); Cognitive complaints: Cognitive Failure Questionnaire (CFQ)* (participant and proxy); participation in everyday life activities: modified version of the Utrechtse Schaal voor Evaluatie en Revalidatie- Participatie (USER-P)* (participant); instrumental activities of daily life: Lawton & Broady Instrumental activity of daily life scale (IADL)* (participant and proxy); and Quality of life: Short Form Health Survey (SF-36)* (participant). These questionnaires are brief and are commonly used in clinical and research settings. Completing these questionnaires will take in total 75 minutes for the participant see appendix 3 for administration occasions) and 35 minutes for the proxy (twice).

* = Online measures

<u>Magnetic Resonance Imaging</u>

Participants will be scanned in the 3T Magnetic Resonance Imaging (MRI) scanner of the Spinoza center. The scan protocol (see appendix 6) is based on sequences frequently used in both Spinoza center and other research centers. The participants will not be required to perform any tasks during the scans. However, one questionnaire will be administered directly after the resting state scan. The total scan time will be approximately 50 minutes. In all scans, a mask of the lesion will be created to assure that this part will not be taken into account during analysis.

Resting state functional MRI

Graph theoretical metrics such as degree distribution as a measure of resilience or robustness (Guye et al., 2010) will be obtained. This measure reflects 'the distribution of the number of connections linking each node to other nodes throughout the network' (Guye et al., 2010). Furthermore, global and local efficiency of information exchange, small-worldness, and centrality will be quantified.

Cardiac and respiratory function, and a questionnaire determining what participants thought about during the resting state scanning, will be obtained as potentially confounding factors. Furthermore, participants will rate whether they fell asleep during resting-state fMRI.

DTI

White matter will be measured with diffusion weighted imaging. DTI measures will be used to assess global and local structural connectivity.

VBM

Gray matter will be measured with T1 weighted structural MRI scans. Voxel based morphometry (VBM) will be used to examine possible gray matter changes due to training. Focus will be on the frontal lobe. Furthermore, lesion characteristics marked by a neuroradiologist such as type of stroke, lesion size and location, diffuse versus local, and size of the damaged network, will be derived from FLAIR and T2 weighted structural MRI scans to examine whether they can predict treatment outcome.

6.1.3. Other parameters

Training measures

During the training the following will be registered automatically by training website or researcher: amount of training actually performed; amount of extra personal (phone or email) contact due to questions during training; and level of engagement (i.e. how often needed to be reminded to train). Furthermore, patients will note in a daily log which takes less than five minutes to complete: level of motivation during training; amount of physical exercise at the day of training; how interesting and difficult were the tasks of that day; and fatigue level before and after training. Once every week or two weeks participants will be asked by phone (see procedures section) the amount of rehabilitation obtained during study (amount of cognitive rehabilitation, if so: occupational rehabilitation, physical rehabilitation). Finally, at T1 and T2 an exit questionnaire will be administered which includes questions about subjective treatment effectiveness measure (confirmation of blindness to condition); change of strategies during training; change in cognitive stimulation in daily life besides study related training; major changes during training (e.g. change of medication).

Stroke measures

During baseline assessment participants will be asked their age at injury, time since injury, cognitive complaints directly after stroke, amount of rehabilitation obtained prior to study, and back to work status. Furthermore, they will rate their recovery after stroke on a 10 cm vertical visual analogue scale.

Mood

Depression will be assessed with the Hospital Anxiety Depression Scale* (HADS; Zigmond & Snaith, 1983). The HADS consists of seven items administering depression and seven items for anxiety. Questions can be answered on a 4-point scale within approximately five minutes. Fatigue will be assessed with the Checklist Individual Strength- Fatigue subscale (CIS-f)*(Vercoulen et al., 1997). This subscale consists of 8 statements that can be answered on a 7-point scale within five minutes.

Demographics and others

Prior to training, cognitive status will be screened with the Telephone Interview Cognitive Status (TICS; Kempen, Meier, Bouwens, van Deursen, & Verhey, 2007). This is a cognitive screening which is administered by phone and

consists of 25 items within approximately 10 minutes. Furthermore, prior to training, visuoperception will be measured with D-KEFS visual scanning condition (Delis, Kaplan, & Kramer, 2001) to assure absence of neglect.

Possible confounders

Educational level will be estimated based on UNESCO ISCED 1997. Comorbidity will be assessed by asking the participant whether they are under medical treatment for anything else than stroke. Moreover, participants will be asked about their possible alcohol and drugs use; occupation; prior neuropsychological assessment, or participation in similar research projects; computer skills and computer aversion; and previous computer game experience.

6.2. Randomisation, blinding, and treatment allocation

Participants will be randomized immediately after TICS has been administered and in- and exclusion criteria have been checked, but before baseline assessments. The minimization technique (Pockock and Simon, 1975) will be used to minimize imbalance between the treatment arms for time since stroke, level of computer experience, and age, scores on TICS, and sex. Indications for breaking the allocation code are not applicable. Both subjects and assessors of cognitive and functional outcome measures will be blind for group allocation.

6.3. Study procedure

Inclusion and training assignment

Clinicians of the rehabilitation center who is treating the patient will select patients who fulfill inclusion criteria from their database via Diagnose Behandeling Combinatie (DBC). They will ask the patient whether he/she is interested in participating in this study. Patients who indicate to be interested will receive written information regarding the study either by post or at one of their treatment sessions. Furthermore, the patient is asked for permission to provide their contact details to the researchers. This may be done already in the acute phase post-stroke. At least one week after patient has provided permission to be contacted by the researcher, patients will be contacted by the researcher to provide further information (if needed) and to invite them to participate**.** Interested patients who are less than 3 months (post-acute study) or 12 months (chronic study) post stroke will be contacted by the researcher as soon as they are 3 or 12 months post stroke.

Whenever patients indicate that they will participate in the study, an online provisional informed consent form will be ‘signed’ and an online screening questionnaire and a cognitive screening by phone (TICS) will be administered to confirm inclusion and exclusion criteria. Permission will be asked to access rehabilitation and hospital files considering the exclusion, inclusion criteria (e.g. presence of cognitive dysfunctions during rehabilitation), tasks used in neuropsychological assessment, and lesion characteristics. Patients will be contacted by phone to confirm whether they can participate in the study.

Those who are included in the study will be randomly assigned to one of the two training conditions, i.e. the cognitive flexibility training condition or the active mock training control group. Both participants and assessors will be kept blind to the training condition. Participants will be told that the study aims to compare two types of cognitive training by computer games, but not that one training is expected to result in superior improvements. Moreover, they are asked not to talk about training content during follow-up assessments to assure that assessors remain blind to training allocation. Likewise, they are asked not to talk about the training sessions with other study participants.

Training instruction and baseline assessment

Participants within the same training condition will be invited together, in pairs of three or four, to the University of Amsterdam (UvA) where a paper version of the informed consent form will be signed after the study is explained. Thereafter, participants will undergo a face-to-face neuropsychological assessment and complete several online tasks (see materials section).

Furthermore, participants will be shown instruction videos of the training tasks on individual computers after which they will practice the tasks. A researcher will be present to explain the training and to answer questions. Participants will be asked to train at moments when they know they have at least 50 minutes available so that they will not be stressed by time pressure. Moreover, they will be asked to train at moments of the day when they are not mentally fatigued (e.g. not to train late at night). Participants will be stimulated to perform well. Both groups will be provided with the same motivational instructions. Furthermore, they will be informed that their training frequency will be monitored. Since performance on training tasks will be analyzed afterwards, it will be emphasized that only the participant himself or herself should use the personal login codes and thus only the participant should do the tasks on his/her own account. Furthermore, the importance to not engage in new cognitively stimulating activities (e.g. start playing bridge, start playing other computer games than the study games, start using game applications on their mobile phones) during the study period will be stressed. Similarly, they are asked to continue with cognitive stimulating activities that were part of their lives already before training. However, use of brain training programs, other than the study training, during study period is not allowed. Therapists will be asked not to use any other computer based cognitive trainings for the duration of the study. The order of assessment relative to instruction will be counterbalanced across participants.

When participants are unable to come to the UvA, assessment will be performed at the home of the participant. Where possible, proxies of the participants will be asked to complete the CFQ, IADL and DEX about the participant. The day before and after the instruction day (this will be counterbalanced) participants will complete the online questionnaires and the online tasks (see materials section) which are thus split into two sessions.

MRI

Before training starts, a selected subset of participants who are able to come to the MRI scanner and who fulfill all MRI inclusion and exclusion criteria will be scanned. Where possible the MRI scan will be combined with one of the days of the online task and questionnaire administration. Participants will be screened with an MRI safety checklist. Then they will come to the Spinoza center where they are prepared for MRI and explained the MRI procedure (see materials section). The questionnaire, which will be administered in the MRI scanner, will be practiced before the participants enter the scanner. When the participants are in the scanner they will be instructed that they can relax or watch a movie (structural part). In the second part (resting-state fMRI) participants will be instructed to close their eyes but not fall asleep. Directly following the resting-state fMRI scan, a questionnaire regarding their thoughts during the resting-state fMRI scan will be administered while participants are in the scanner. The total scan procedure will take one hour. MRI images will be assessed for abnormalities by a neuroradiologist. In case of a treatable incidental finding the general practitioner of the participant will be informed and asked to contact the participant about this matter.

<u>Training</u>

Training sessions will be completed at home on the online website five times per week for half an hour. Once per week or two weeks (week 1, 2, 3, 5, 6, 8, and 10), personal feedback will be provided by phone, and the participants are given the opportunity to ask questions. Participants are motivated to write down all questions related to the training tasks. Furthermore, participants will hold a daily log in which they mark their level of motivation, fatigue, and amount of physical exercise performed that day. Within this log they are given the possibility to provide feedback related to training, questions, and feelings about their performance. When participant have urgent questions regarding training they can call the researcher who provided the instructions at the first session. Similarly, when the researcher notices from the log file that the participant is having difficulties with a training task, the participant will be contacted to clarify instructions. Participants will be sent a reminder email to train when they did not train for two days within one week. When participants did not train for three days within a week, they will be called by the researcher and will be reminded to train.

<u>Assessments at different time points</u>

After 6 weeks, a subset of neuropsychological tasks and questionnaires, assessed before training, will be repeated online. These tasks will be done at home instead of one training session. Participants will complete the first part of the exit questionnaire. After the 12 weeks of training sessions, participant will be invited to come to the UvA where they will again be assessed with the same tasks and questionnaires as prior to training. When participants were assessed at home prior to training they will again be administered at home on the same computer. Furthermore, the second exit questionnaire will be administered. Proxies will be asked to complete the same questionnaires about the participant. Those who had an MRI scan prior to training are again scanned post-training. In case of early termination of training by the participant, post testing is performed directly after training

termination (provided that the patient is still willing to participate in the assessments). One month after training completion, participants are asked to complete the online neuropsychological tasks and questionnaires at home. If necessary, participant will be reminded by email and phone to complete the assessment session. When the participant is interested, lifestyles tips for increasing likelihood of successful aging will be provided at the end of the study.

In total the time investment for this study (including assessments and training) is approximately 50 hours per participant, spread over four months. Total duration of all assessment moments can be found in appendix 3.

Treatment compliance will be checked by visual inspection of the training log created by the training website.

6.4. Withdrawal of individual subjects

Subjects can leave the study at any time for any reason if they wish to do so without any consequences.

6.5. Replacement of individual subjects after withdrawal

All participants (including those who withdraw after commencement of the training) will be included in the intention to treat analysis. If time and resources permit, new participants will be recruited to supplement for participants who dropped out.

6.6. Follow-up of subjects withdrawn from treatment

Care as usual will be given to patients who decide to withdraw from the study.

6.7. Premature termination of the study

We do not expect any harmful effects of our training. Therefore, criteria for terminating the study prematurely are not applicable.

7. SAFETY REPORTING

There are no foreseeable safety risks in this study. Nonetheless, we will adhere to the following conventions.

7.1. Section 10 WMO event

In accordance to section 10, subsection 1, of the WMO, the investigator will inform the subjects and the reviewing accredited METC if anything occurs, on the basis of which it appears that the disadvantages of participation may be significantly greater than was foreseen in the research proposal. The study will be suspended pending further review by the accredited METC, except insofar as suspension would jeopardise the subjects' health. The investigator will take care that all subjects are kept informed.

7.2. Adverse and serious adverse events

Adverse events are defined as any undesirable experience occurring to a subject during the study, whether or not considered related to [the investigational product / the experimental treatment]. All adverse events reported spontaneously by the subject or observed by the investigator or his staff will be recorded.

A serious adverse event (SAE) is any untoward medical occurrence or effect that at any dose:

- results in death;
- is life threatening (at the time of the event);
- requires hospitalisation or prolongation of existing inpatients' hospitalisation;
- results in persistent or significant disability or incapacity;
- is a congenital anomaly or birth defect;
- is a new event of the trial likely to affect the safety of the subjects, such as an unexpected outcome of an adverse reaction, lack of efficacy of an IMP used for the treatment of a life threatening disease, major safety finding from a newly completed animal study, etc.

There are no known or expected serious adverse events for the current study. All SAEs will be reported through the web portal *ToetsingOnline* to the accredited METC that approved the protocol, within 15 days after the sponsor has first knowledge of the serious adverse reactions.

SAEs that result in death or are life threatening should be reported expedited. The expedited reporting will occur not later than 7 days after the responsible investigator has first knowledge of the adverse reaction. This is for a preliminary report with another 8 days for completion of the report.

7.3. Follow-up of adverse events

All adverse events will be followed until they have abated, or until a stable situation has been reached. Depending on the event, follow-up may require additional tests or medical procedures as indicated, and/or referral to the general physician or a medical specialist.

8. STATISTICAL ANALYSIS

8.1. Descriptive statistics

Analyses will be performed to evaluate the comparability of the intervention and the active control group at baseline on sociodemographic and clinical variables. Depending on the level of measurement a Student's t-test or non-parametric statistics will be used. If group differences are found on one or more variables that might be confounded with the treatment effect, those variables will be included in further analyses as covariates.

8.2. Primary and secondary analysis

Primary analyses

The difference in training effects on the dependent variables between the two stroke samples will be analyzed with a Student's t test. When there is no difference between stroke groups they will be pooled.

First, in an intention to treat analysis linear mixed models with group and group by time interaction as factors will be performed. Covariates will be age, education, computer skills prior to training, stroke severity, TICS score. The dependent variables will be the primary outcome measure; objective executive functioning difference compared to baseline. Normal distribution will be checked by Kolmgorov–Smirnov tests. Values smaller than $p = .05$ will be considered significant. Second, per protocol analyses will be performed. All analysis will be repeated with the adherence only sample (those who adhered to full study protocol) which will also exclude participants who suffered from a major medical event (such as recurrent stroke, seizure, major depression) during the study.

Secondary analyses

First, above mentioned analyses will also be conducted for the secondary measures: change from baseline of cognitive flexibility; change from baseline of cognitive function; training improvement; and change from baseline of subjective cognitive and everyday life functioning. Second, imaging analyses will be performed. Independent component analysis will be used to extract networks of brain activity from resting-state MRI (Beckmann, DeLuca, Devlin, & Smith, 2005). Dual regression will be used to analyze these data. DTI data will be analysed using tract-based spatial statistics (Smith et al., 2006). Gray matter will be analyzed by voxel based morphometry (VBM) (Ashburner & Friston, 2000). Student's t-test will be used to analyze changes from baseline after training differences between intervention and active control group of: the values derived from DTI analyses, VBM values, graph metrics. Additionally, analyses which take compliance and rehabilitation therapy into account will be carried out.

We will explore whether the effectiveness of cognitive flexibility training varies as a function of baseline executive functioning, health state (stroke versus healthy) and time since stroke (sub-acute versus chronic). Linear regressions will be used to determine predictor variables of good training outcome. Finally, proxy measures will be compared with those of the participants.

9. ETHICAL CONSIDERATIONS

9.1 Regulation statement

This study will be conducted according to the principles of the Declaration of Helsinki (version 6, 2008) and in accordance with the Medical Research Involving Human Subjects Act (WMO).

9.2. Recruitment and consent

Patients will be recruited from patient databases of rehabilitation centers (e.g. Reade and Heliomare). Those who fulfill inclusion criteria will be asked by a clinician to participate in this study. Patients will be asked directly, or via their caregiver, or will be sent a letter with a request to participate in this study. The participant may be informed about the study by the clinician earlier than 3 or 12 months post-stroke.

Patients who indicate to be interested will receive written information regarding the study either by post or at one of their treatment sessions. Furthermore, the patient is asked for permission to provide their contact details to the researchers. After at least one week, patients will be contacted by the researcher to provide further information (if needed) and to ask whether they are willing to participate**.** Interested patients who are less than 3 months (post-acute study) or 12 months (chronic study) post stroke will be contacted by the researcher as soon as they are 3 or 12 months post stroke.

Participants will also be recruited by advertisements in newsletters of Dutch stroke patients associations. The advertisement will call for people who suffered from mild to moderate stroke less than three years ago, who received rehabilitation and who have remaining cognitive complaints. Those interested in participating in our study will be asked to contact the researchers. This procedure is equal to the pilot study which was approved by the ethical committee of the Faculty of Social and Behavioural Sciences of the University of Amsterdam.

Everyone fulfilling inclusion and exclusion criteria and interested in participating in this study will receive written information about this project. This information will state the time investment needed, in- and exclusion criteria and the assessment procedure. Participants will not be told that it is expected that one of the conditions will result in superior improvements. They will be told that the study will examine training effects of two different trainings. People who are willing to participate are asked to fill out an informed consent form. This form states that anonymity will be guaranteed and that they can decide to withdraw from the intervention program at any time without any consequences.

9.3. Benefits and risks assessment, group relatedness

Participants will have to train at least 30 minutes per day for five days per week for 12 weeks. Furthermore, participants will be assessed twice (1 hour per assessment at UvA), perform several computer tasks (2 hours at home) four times, questionnaires (1 hour at home), and receive information about the training (1 hour at UvA). In total, including 60 training sessions, participants will spend 50 hours on this study. Nevertheless, we expect that participants will not find the training burdensome because it is designed to be interesting and challenging. Participants who participate in the MRI part of the study will also be scanned twice for one hour. To our knowledge there are no risks involved. Difference in benefit is expected between the flexibility training and the active control condition. However, so far none of the treatments has been proven to be superior to the other**.** Moreover, both groups will be offered the opportunity to train with the flexibility tasks used in the intervention group after the study period.

It is expected that intensive computer based cognitive flexibility training will result in improvement of executive functioning. Patients may benefit from this in their everyday life activities. Furthermore, results of this study can be helpful for development of treatments that can assist the recovery of people suffering from stroke. Risks of

computer-based training are low. These potential benefits and the limited risks justify the implementation of the study.

9.4. Compensation for injury

Dispensation from the statutory obligation to provide insurance for the participants in this study will be requested. This study protocol does not carry risks for the participants and, therefore, we believe that a dispensation should be granted.

9.5. Incentives

The participant will receive compensation for their travel costs (max two times 20 euros). Furthermore, the participant will be offered a small present and a unlimited, free subscription to Braingymmer. Participants participating in the MRI part of the study will receive 15 euro's per MRI scan (max two times 15 euros).

10. ADMINISTRATIVE ASPECTS AND PUBLICATION

10.1 Handling and storage of data and documents

Data will be handled anonymously. The principal investigator (prof. dr. J.M.J. Murre), Prof dr. B. Schmand, and an external person who performed the randomization coding procedure will have access to the code. The principal investigator will safeguard the key to the code. Data will be kept for 15 years and will be stored according to the 'Wet medisch wetenschappelijk onderzoek met mensen' (WMO) and 'Wet op de geneeskundige behandelovereenkomst' (WGMO).

Privacy sensitive information, including test data from all neuropsychological tests, received from home computers will be transmitted with a secure http (https) protocol and stored in a secure database that cannot be accessed directly from the outside (Qualtrics database, UvA Lotus database, NeuroTask database). No data will be stored on the subject's home computers. In the database, subjects are identified only with a number without any data with which they could be identified (e.g., exact age). Any such privacy-sensitive data will be stored in a secure, physically separate location by the two graduate researchers of this project and protected with a password. Thus, even if any of the databases would be hacked, which is highly unlikely, it will be impossible to link any of the data to a subject's identity.

10.2 Amendments

Amendments are changes made to the research after a favourable opinion by the accredited METC has been given. All amendments will be notified to the METC that gave a favourable opinion.

10.3. Annual progress report

The sponsor/investigator will submit a summary of the progress of the trial to the accredited METC once a year. Information will be provided on the date of inclusion of the first subject, numbers of subjects included and

numbers of subjects that have completed the trial, serious adverse events/ serious adverse reactions, other problems, and amendments.

10.4. End of study report

The investigator will notify the accredited METC of the end of the study within a period of 8 weeks. The end of the study is defined as the last participant's last measurement. In case the study is ended prematurely, the investigator will notify the accredited METC, including the reasons for the premature termination. Within two years after the end of the study, the investigator/sponsor will submit a final study report with the results of the study, including any publications/abstracts of the study, to the accredited METC.

10.5. Public disclosure and publication policy

No restrictions regarding the public disclosure and publication of the research have been or will be made by the sponsor.

References

Ashburner, J., & Friston, K. J. (2000). Voxel-based morphometry - the methods. *NeuroImage, 11*(6) doi: 10.1006/nimg.2000.0582

Beckmann, C. F., DeLuca, M., Devlin, J. T., & Smith, S. M. (2005). Investigations into resting-state connectivity using independent component analysis.*360*, 1001-1013. doi: 10.1098/rstb.2005.1634

Belleville, S., Clement, F., Mellah, S., Gilbert, B., Fontaine, F., & Gauthier, S. (2011). Training-related brain plasticity in subjects at risk of developing alzheimer's disease. *Brain, 134* doi: 10.1093/brain/awr037

Buitenweg, J. I. V., Murre, J. M. J., & Ridderinkhof, K. R. (2012). Brain training in progress: A review of trainability in healthy seniors. *Frontiers in Human Neuroscience, 6*, 183. doi: 10.3389/fnhum.2012.00183

Culbertson, W., Zillmer, E. (2005). *Tower of London Drexel University*. Chicago: Multi- Health Systems.

Dahlin, E., Neely, A. S., Larsson, A., Backman, L., & Nyberg, L. (2008). Transfer of learning after updating training mediated by the striatum. *Science, 320*(5882), 1510-1512. doi: 10.1126/science.1155466

Delis, D. C., Kaplan, E., & Kramer, J. (2001). *Delis–Kaplan executive function system*. San Antonio, TX: Psychological Corporation.

Johansson, B., & Tornmalm, M. (2012). Working memory training for patients with acquired brain injury: Effects in daily life. *Scandinavian Journal of Occupational Therapy, 19*(2), 176-183. doi: 10.3109/11038128.2011.603352

Karbach, J., & Kray, J. (2009). How useful is executive control training? age differences in near and far transfer of task-switching training. *Developmental Science, 12*(6), 978-990. doi: 10.1111/j.1467-7687.2009.00846.x

Kempen, G. I. J. M., Meier, A. J. L., Bouwens, S. F. M., van Deursen, J., & Verhey, F. R. J. (2007). [The psychometric properties of the dutch version of the telephone interview cognitive status (TICS)]. [Telefonisch Interview Cognitieve Status (TICS): psychometrische aspecten.] *Tijdschrift Voor Gerontologie En Geriatrie, 38*(1)

Madureira, S., Guerreiro, M., & Ferro, J. M. (2001). Dementia and cognitive impairment three months after stroke. *European Journal of Neurology, 8*(6), 621-627. doi: 10.1046/j.1468-1331.2001.00332.x

Mozolic, J. L., Hayasaka, S., & Laurienti, P. J. (2010). A cognitive training intervention increases resting cerebral blood flow in healthy older adults. *Frontiers in Human Neuroscience, 4*, 16.

Owen, A. M., Hampshire, A., Grahn, J. A., Stenton, R., Dajani, S., Burns, A. S., . . . Ballard, C. G. (2010). Putting brain training to the test. *Nature, 465*(7299) doi: 10.1038/nature09042

Peretz, C., Korczyn, A. D., Shatil, E., Aharonson, V., Birnboim, S., & Giladi, N. (2011). Computer-based, personalized cognitive training versus classical computer games: A randomized double-blind prospective trial of cognitive stimulation. *Neuroepidemiology, 36*(2) doi: 10.1159/000323950

Raven, J. C. (1995). *Manual for the coloured progressive matrices (revised)*. Windsor, UK: NFRE-Nelson.

Rogers, R. D., & Monsell, S. (1995). Costs of a predictable switch between simple cognitive tasks. *Journal of Experimental Psychology-General, 124*(2) doi: 10.1037//0096-3445.124.2.207

Schmand, B., Groenink, S. C., & van de Dungen, M. (2008). Letterfluency: Psychometrische eigenschappen en nederlandse normen. *Tijdschrift Gerontol Geriat, 39*, 64-76.

Smith, S. M., Jenkinson, M., Johansen-Berg, H., Rueckert, D., Nichols, T. E., Mackay, C. E., . . . Behrens, T. E. J. (2006). Tract-based spatial statistics: Voxelwise analysis of multi-subject diffusion data. *NeuroImage*, *31*(4) doi: 10.1016/j.neuroimage.2006.02.024

Stablum, F., Umilta, C., Mazzoldi, M., Pastore, N., & Magon, S. (2007). Rehabilitation of endogenous task shift processes in closed head injury patients. *Neuropsychological Rehabilitation, 17*(1) doi: 10.1080/13506280500411111

Tatemichi, T. K., Desmond, D. W., Stern, Y., Paik, M., Sano, M., & Bagiella, E. (1994). Cognitive impairment after stroke - frequency, patterns, and relationship to functional abilities. *Journal of Neurology Neurosurgery and Psychiatry, 57*(2), 202-207. doi: 10.1136/jnnp.57.2.202

Vercoulen, J. H. M. M., Bazelmans, E., Swanink, C. M. A., Fennis, J. F. M., Galama, J. M. D., Jongen, P. J. H., . . . Bleijenberg, G. (1997). Physical activity in chronic fatigue syndrome: Assessment and its role in fatigue. *Journal of Psychiatric Research, 31*(6) doi: 10.1016/S0022-3956(97)00039-3

Wechsler, D. (2000). *Wechsler Adult Intelligence Scale (WAIS-III) Nederlandstalige bewerking. Technische handleiding*. Lisse: Swets & Zeitlinger.

Westerberg, H., Jacobaeus, H., Hirvikoski, T., Clevberger, P., Ostensson, M. -., Bartfai, A., & Klingberg, T. (2007). Computerized working memory training after stroke - A pilot study. *Brain Injury, 21*(1) doi: 10.1080/02699050601148726

Zachary, R. A. (1991). *Shipley institute of living scale: Revised manual*. Los Angeles: Western Psychological Services.

Zigmond, A. S., & Snaith, R. P. (1983). The hospital anxiety and depression scale. *Acta Psychiatrica Scandinavica, 67*(6) doi: 10.1111/j.1600-0447.1983.tb09716.x

Dr. H.Steven Scholte, versie 4.1, 23-10-2010

Screening MRI proefpersonen

Appendix 1. MRI screening form

Naam:
Geboortedatum:
Gewicht (schatting):
Adres:

Postcode:
Woonplaats:
Telefoonnummer:
E-mail:

SC Nummer:

Mogen wij in de toekomst contact met U opnemen voor andere onderzoeken?

Ja / Nee

Gebruikt u op dit moment psychofarmaca? ja / nee
Bent u kleurenblind? ja / nee
Draagt u een bril of contactlenzen ja / nee
Zo ja, wat voor afwijking in de diepte? Links = Rechts =

Heeft u of draagt u:
- een pacemaker of (oude) pacemakerdraden? ja / nee
- een medicijnpomp (b.v. insulinepomp)? ja / nee
- een neuro-stimulator? ja / nee
- een uitwendige prothese (b.v. kunstarm)? ja / nee
- één of meerdere piercings op uw lichaam? ja / nee
- tatoeages of permanente make-up? ja / nee
- tandtechnische constructies (beugels, draadjes e.d.)? ja / nee
- medicijnpleisters (nicotine-, hormoonpleisters e.d.)? ja / nee

Heeft u ooit een operatie ondergaan aan:
- het hoofd (b.v. plaatsen vaatclip of pompje)? ja / nee
- het hart (b.v. kunstklep)? ja / nee
- de ogen (b.v. geïmplanteerde lenzen)? ja / nee
- de oren (gehoorbeentjesprothese; hoorapparaat)? ja / nee
- de botten (waarbij platen en schroeven zijn gebruikt)? ja / nee
- anderszins? ja / nee

zo ja, aan ....
.
Bent u (oud) metaalbewerker? ja / nee
Bestaat er kans op metaalsplinters in de oogkas? ja / nee
Heeft u last of ooit last gehad van:
- engtevrees/claustrofobie (b.v. bent u angstig in een lift)? ja / nee
- kortademigheid (bij plat liggen)? ja / nee

Zou u zwanger kunnen zijn? ja / nee

Aldus naar waarheid ingevuld

....
datum handtekening

Appendix 2. Description of training tasks

The training can be reviewed on www.braingymmer.nl

Cognitive flexibility training
All tasks consist of 20 levels.

Attention tasks

Pattern Matrix

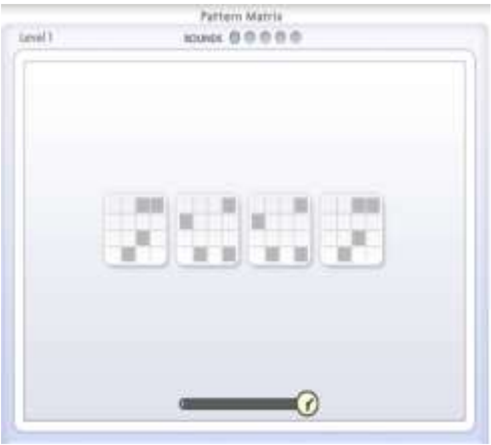

Participants have to mentally rotate patterns to find pairs. This task is under time pressure. The higher the level, the more difficult the patterns become and more patterns will be presented.

Birds of a Feather

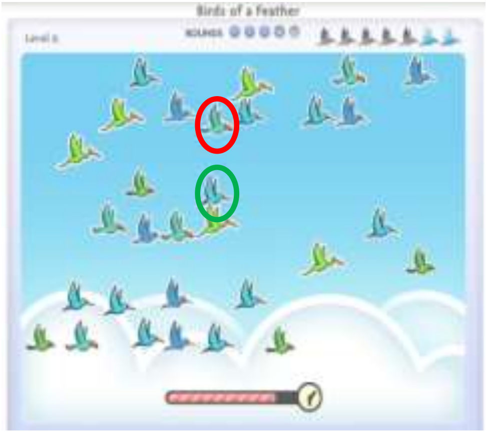

Participants have to count blue birds under time pressure. The higher the level, the more similar the distractor and the to be counted birds become.

Brainfreeze

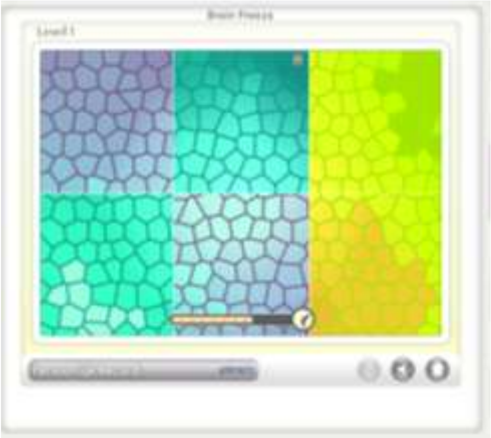

Squares are presented which continuously change colors. Participants have to freeze the squares as soon as they match. More squares in smaller size will be presented in higher levels.

Reasoning tasks

Square Logic

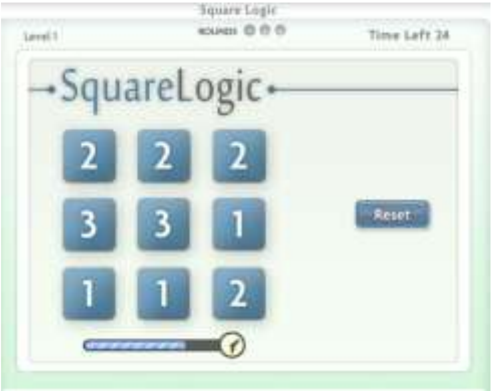

Blocs have to be placed on top of each other (the bottom block will disappear) such that only one block will remain. Blocs can only be placed on blocks which are next to them and are exactly one number higher or lower. This task is under time pressure. Higher levels will include more squares and more numbers.

Out of Order

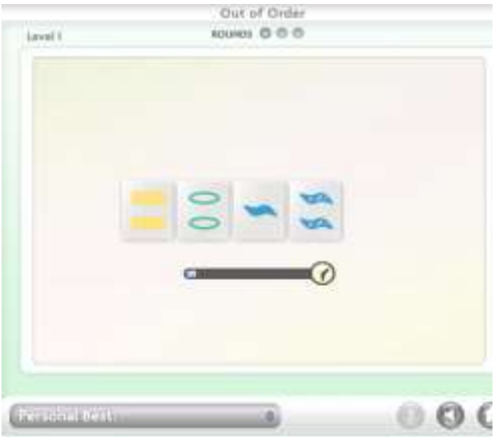

Figures need to be arranged in such a way that they match with their neighbor figure at least on one characteristic. Characteristics are color, shape, filling, and number of figures. This task is under time pressure and will have more tiles at higher levels.

Patterned Logic

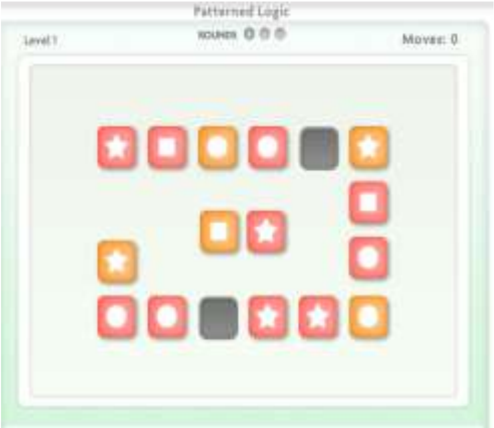

A pattern is shown which is build up out of a color pattern and a figure pattern. Participants have to complete the missing tiles in the pattern. Higher levels will have more gaps in the pattern.

Toy Shop

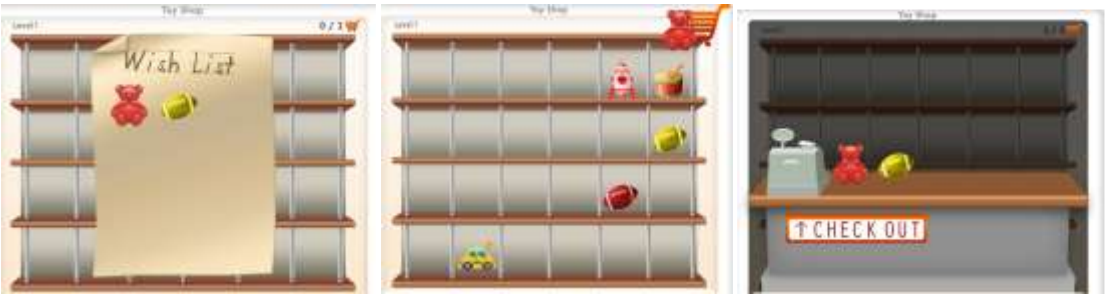

Participants have to remember items from a shopping list and collect these items out of a store.

Multi Memory

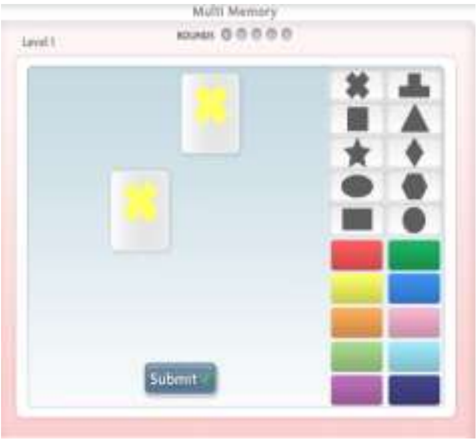

Several figures will be shown in different colors and shapes. The participants have to reconstruct these figures after they have disappeared.

Moving Memory

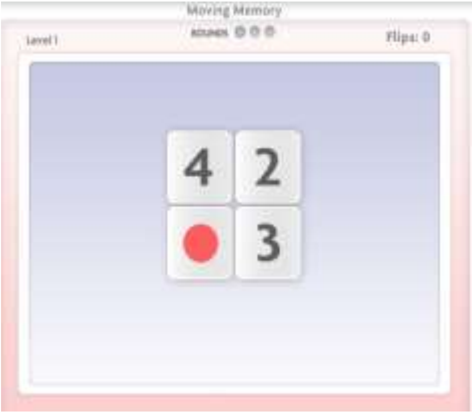

Participants have to find pairs. However, tiles change position after a pair has been found. Thus, figures can only be remembered based on the numbers on the tiles.

<u>Control training</u>
All tasks consist of five levels.

Fuzzle

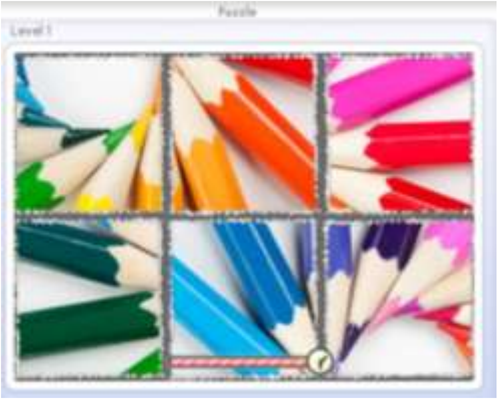

Participants have to reconstruct a fractured picture.

Sliding Search

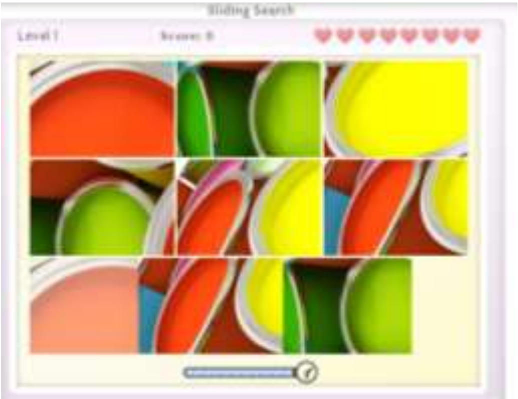

Pictures will slide over the lower part of the screen. Participants need to match pictures from the upper part of the screen with those in the lower part of the screen.

Pay attention

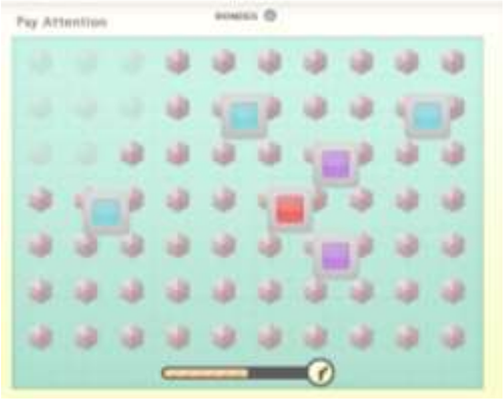

Squares will appear on a screen. Whenever they change color the participant has to click on it.

Grid Tracks

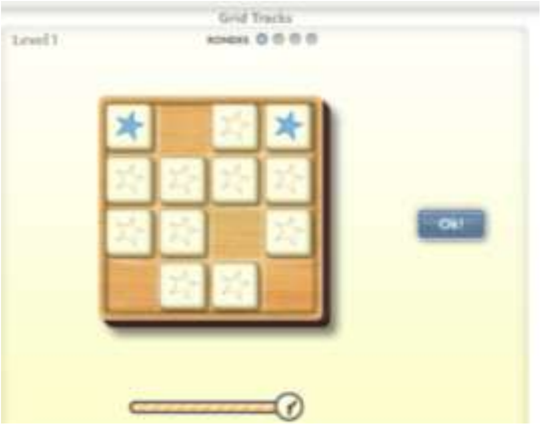

Participants have to mentally follow the trajectory of a couple of blocks which initially have blue stars. When blocks stop moving, participants need to locate the blocks which initially had blue stars.

Appendix 3. Measures at different time points

T=0	T=1	T=2	T=3
3 sessions of 2 hours (incl. 1h MRI & training instructions) divided over three days + one session of 30 min + 25 minutes for proxy	2.2 hours	3 sessions of less than 2 hours (incl. 1h MRI) divided over three days + 25 minutes for proxy	1.5 hours
Primary outcome measures			
D-Kefs TMT (1-5)		D-Kefs TMT (2,4,5)	
ToL (online) Kralen puzzel	ToL (online) Kralen puzzel	ToL (online) Kralen puzzel	ToL (online) Kralen puzzel
Letter-number sequencing		Letter-number sequencing	
Fluency		Fluency	
Secondary outcome measures			
Switching +dual task (online)	Switching task (online)	Switching +dual task (online)	Switching task (online)
TMT (online) Spoorzoeken	TMT (online) Spoorzoeken	TMT (online) Spoorzoeken	TMT (online) Spoorzoeken
DSST (online)	DSST (online)	DSST (online)	DSST (online)
RAVLT (online)		RAVLT (online)	
O-span (online)		O-span (online)	
N-back (online)		N-back (online)	
Corsi block Blokkenreeksen (online)	Corsi block Blokkenreeksen(online)	Corsi block Blokkenreeksen(online)	Corsi block Blokkenreeksen(online)
Pegboard (online) Vlakken vullen	Pegboard (online) Vlakkenvullen	Pegboard (online) Vlakken vullen	Pegboard (online) Vlakken vullen
CPM (online)		CPM (online)	CPM (online)
Verbal reasoning (online)		Verbal reasoning (online)	
PASAT		PASAT	
Go-no-go		Go-no-go	
MRI		MRI	
DEX (online)	DEX (online)	DEX (online)	DEX (online)
CFQ (online)	CFQ (online)	CFQ (online)	CFQ (online)
USER-P (online)		USER-P (online)	USER-P (online)
SF-36 (online)		SF-36 (online)	SF-36 (online)
IADL (online)		IADL (online)	IADL (online)
HADS (online)		HADS (online)	
CIS-F (online)		CIS-F (online)	
Proxy CFQ (online)		Proxy CFQ (online)	
Proxy DEX (online)		Proxy CFQ (online)	
Proxy IADL (online)		Proxy IADL (online)	
TICS			
Demographic questions (online)			
Computer skills (online)		Computer skills (online)	Computer skills (online)
	Subjective Training success (online)	Subjective Training success (online)	
Recovery VAS		Recovery VAS	
Measures for another study but not used in current study			
	Reaction time task Reactietijden		
	Willekeurig klikken		

	Digit span (forward & backward)		
	MVI-20		
	HADS		
	RAVLT (online) Woorden leren		
	= at home		

Note: several measures are administered at T1 for another study and are not analyzed within the current study.

Abbreviation	Measure
D-Kefs TMT	Delis- Kaplan Executive Function System
ToL	Tower of London (Kralen puzzle)
TMT	Trail Making Test (Spoorzoeken)
DSST	Digit Symbol substitution Task
RAVLT	Rey Auditory Verbal Learning Task
O-span	Operation-span
CPM	Raven Coloured Progressive Matrices
PASAT	Pased Auditory Serial Addition Test
MRI	Magnetic Resonance Imaging
DEX	dysexecutive questionnaire
CFQ	Cognitive Failure Questionnaire
USER-P	Utrechtse Schaal voor Evaluatie en Revalidatie-Participatie
SF-36	Short Form Health Survey
IADL	Instrumental activity of daily life scale
HADS	Hospital Anxiety Depression Scale
CIS-F	Checklist Individual Strength- Fatigue subscale
TICS	Telephone Interview Cognitive Status

Appendix 4. Description and duration of measures.

Measure	Cognitive domain	duration
Delis- Kaplan Executive Function System	Executive function	6
Tower of London (Kralen puzzle)	Executive function	10
Letter-number sequencing	Executive function	5
Fluency	Executive function	9
Switching +dual task	Executive function	30
Trail Making Test (Spoorzoeken)	Executive function	10
Digit Symbol substitution Task	Processing speed	5
Rey Auditory Verbal Learning Task	Memory	20
Operation-span	Working memory	20
N-back	Working memory	15
Corsi block Blokkenreeksen	Working memory	5
Pegboard Vlakken vullen	Psychomotor speed	3
Raven Coloured Progressive Matrices	Reasoning	20
Verbal reasoning	Reasoning	10
Pased Auditory Serial Addition Test	Working memory/ attention	10
Go-no-go	Inhibition	5
Questionnaires		
DEX (online)	Executive function	20
CFQ (online)	General cognitive function	10
USER-P (online)	Participation	15
SF-36 (online)	Quality of life	15
IADL (online)	IADL	5
HADS (online)	Depression/anxiety	5
CIS-F (online)	Fatigue	5
Proxy CFQ (online)	General cognitive function	10
Proxy DEX (online)	Executive function	20
Proxy IADL (online)	IADL	5
TICS	General cognitive function	10
Recovery VAS		1

Appendix 5. Composite scores of several cognitive domains

Attention

- Trail making test contrast condition B corrected for A (TMT;UVA Neurotest BV)
- Paced Auditory Serial Addition Task * (PASAT; Gronwall, 1977)
- Digit symbol substitution task * (DSST; WAIS III-NL; Wechsler, 2000)

Memory

- Rey's auditory verbal learning test * (RAVLT; Saan & Deelman, 1986)

Working Memory

- O-span *
- N-back *(UvA Neurotest BV)
- Blokkenreeksen (UvA Neurotest BV); online modified version of corsi block task *

Intelligence

- Raven Coloured Progressive Matrices * (CPM; (Raven, 1995))
- Shipley Institute of Living Scale-2 *(Zachary, 1991)

Psychomotor speed

- D-Kefs TMT motor speed condition (Delis, Kaplan & Kramer, 2007)
- Vlakken vullen (UvA Neurotest BV); online modified version of Pegboard *

Inhibition

- go-no-go task

* = Online measures

Appendix 6. Scan protocol

1) **SmartBrain** Duration: 53 seconds.	Filmpje
2) **RefScan4** Duration 59 seconds.	Filmpje
3) **T1** (**sT13DTFE_P25_S2_6m**) Duration: 6 minutes	Filmpje
4) **B0** Duration 108 seconds	Filmpje
5) **Resting-state EPI (TRA_3mm_ISO)** Duration: 10 minutes (300 volumes)	Instructie + Lampen uit + beamer uit/ zwart scherm (begin van Himalaya)
6) Questionnaire thoughts during RS	Vragenlijst + in slaap gevallen?
7) High resolution T2 (T2w, isotrope 1 mm resolutie) ongeveer 5 min	Filmpje
8) **RefScan4** Duration 59 seconds. (before every SENSE scan)	Filmpje
**9) 3x DWI (3x DTI_FA)** Duration 4.36 x 3 = 13.08 minutes	Filmpje
**10)** T2 FLAIR (toevaltreffer) ongeveer 3 min	Filmpje
